# Supplementary figures and images for: IUSMMT: Survival mediation analysis of gene expression with multiple DNA methylation exposures and its application to cancers of TCGA
Source: PLoS Comput Biol. 2021 Aug 31;17(8):e1009250. doi: 10.1371/journal.pcbi.1009250 (PMC8437300; doi:10.1371/journal.pcbi.1009250)

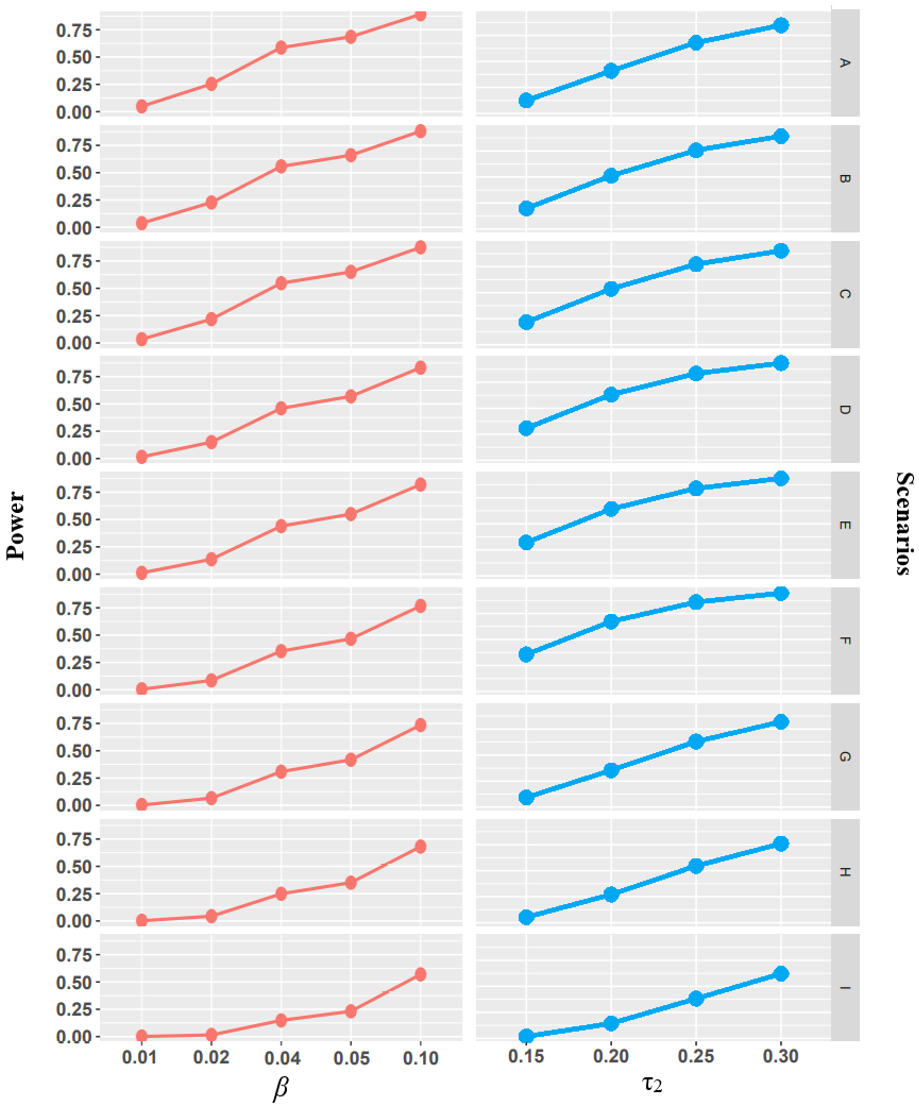

Supplement: S1 Fig — These powers are estimated under six alternative simulation scenarios from A to I with various mixture proportions. The graph in the left column is the power of β, and the x-axis is the value of β = 0.15, 0.20, 0.25 or 0.30; The graph in the left column is the power of α, and the x-axis is the value of τ2 = 0.01, 0.02, 0.04, 0.05 or 0.10. These powers are estimated by the average across the 100 replications. (TIF) [file pcbi.1009250.s001.tif]

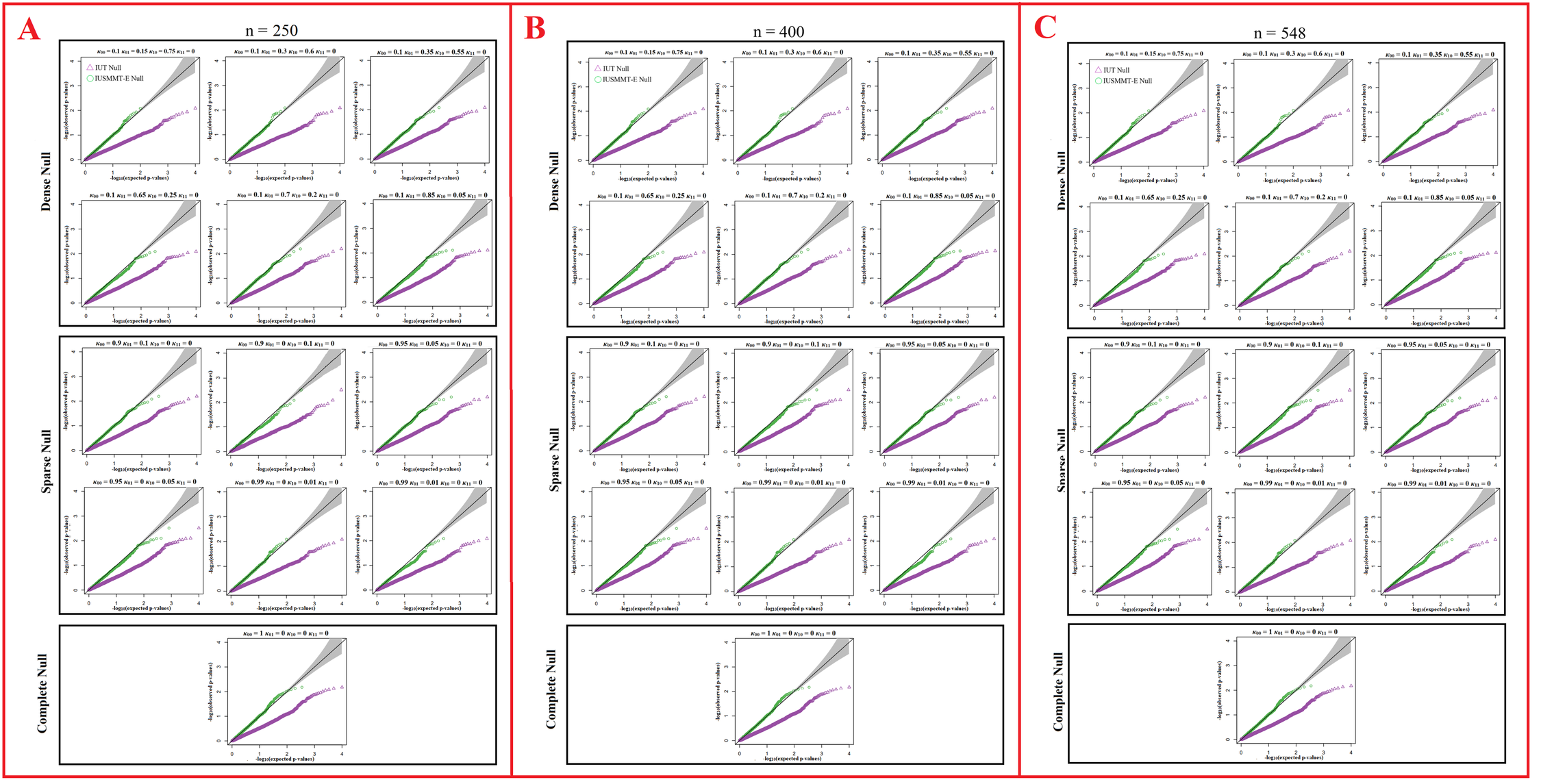

Supplement: S2 Fig — Here (A), (B) and (C) represent the sample size n = 250, 400 and 548, respectively. (TIF) [file pcbi.1009250.s002.tif]

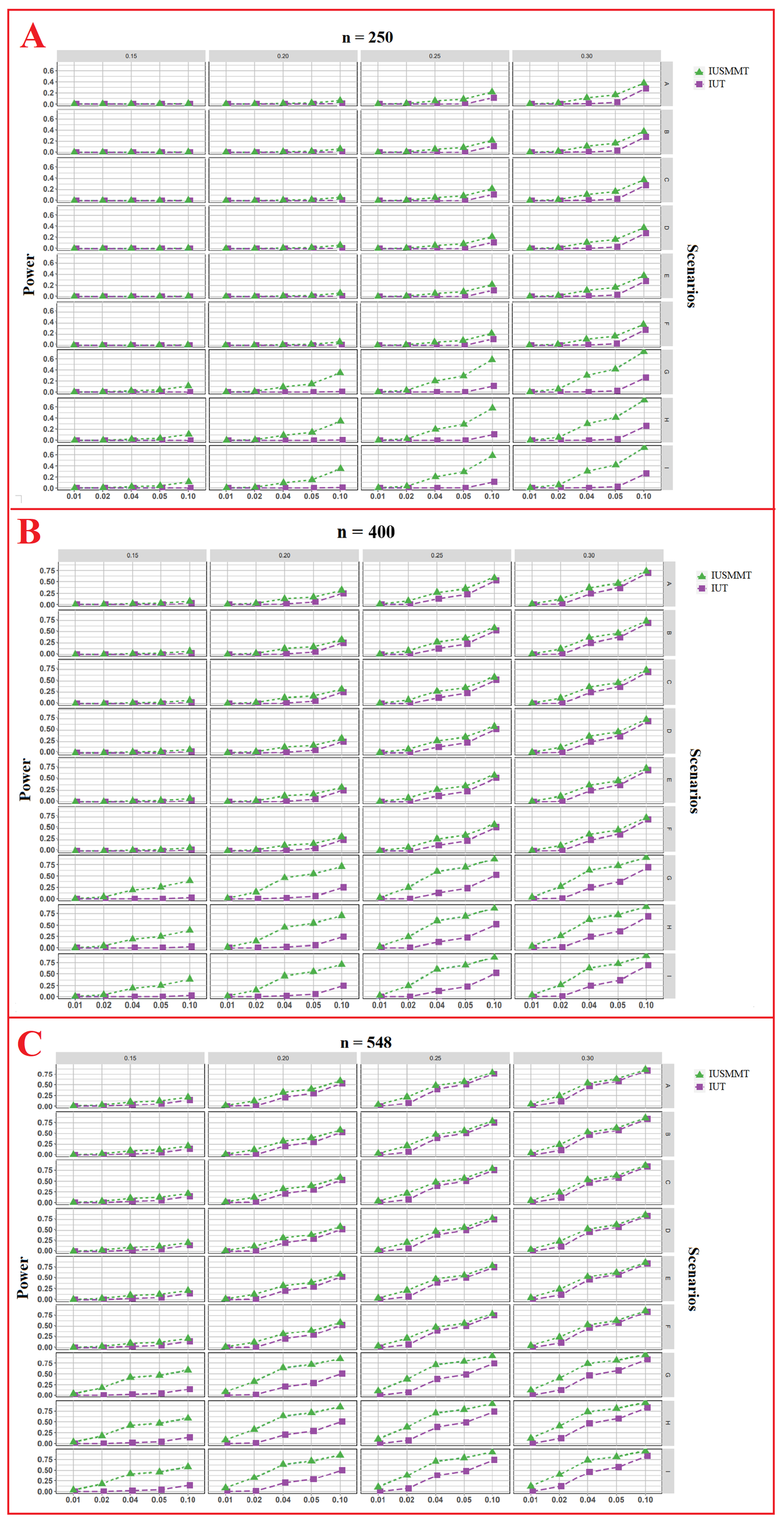

Supplement: S3 Fig — Here, τ2 = 0.01, 0.02, 0.04, 0.05 or 0.10 at the x-axis, β = 0.15, 0.20, 0.25 or 0.30 on the top. These powers are estimated for the nine alternative simulation scenarios from A to I with various values for the mixture proportions by the average across the 100 replications. (A), (B) and (C) represent the sample size n = 250, 400 and 548, respectively. (TIF) [file pcbi.1009250.s003.tif]

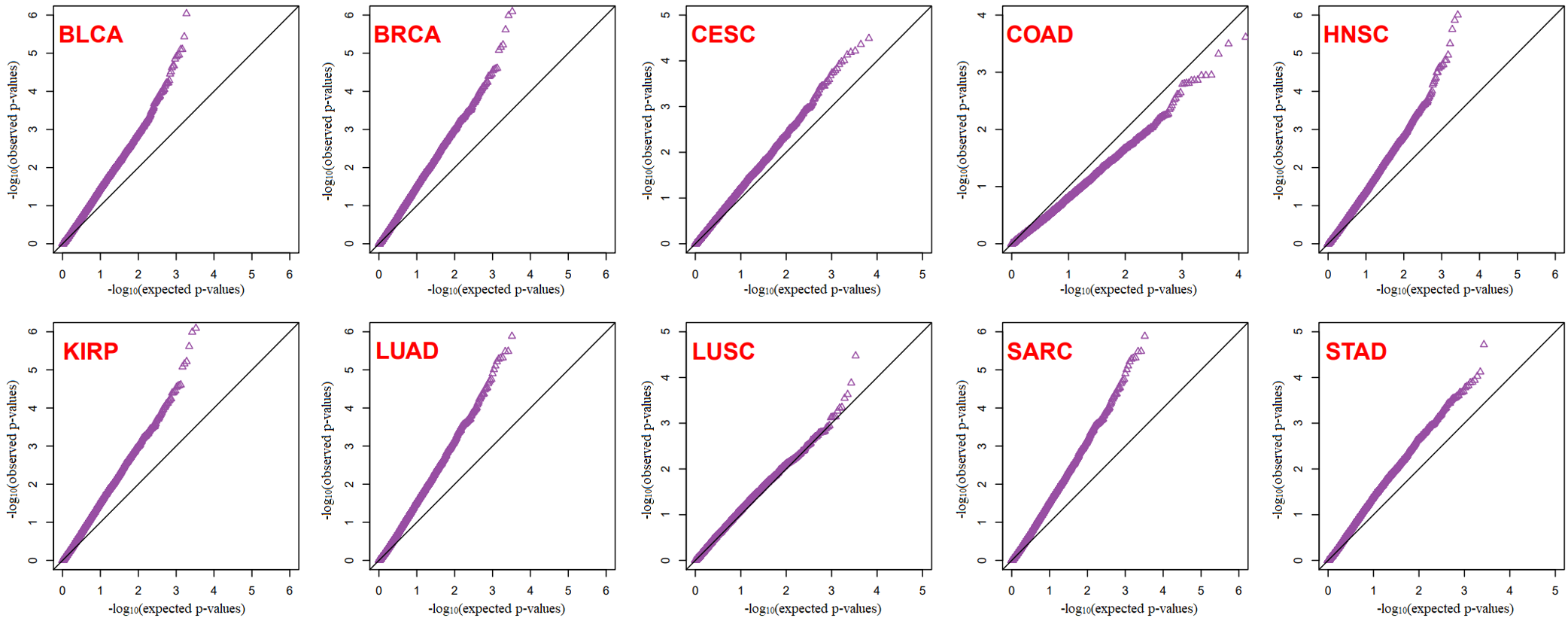

Supplement: S4 Fig — (TIF) [file pcbi.1009250.s004.tif]

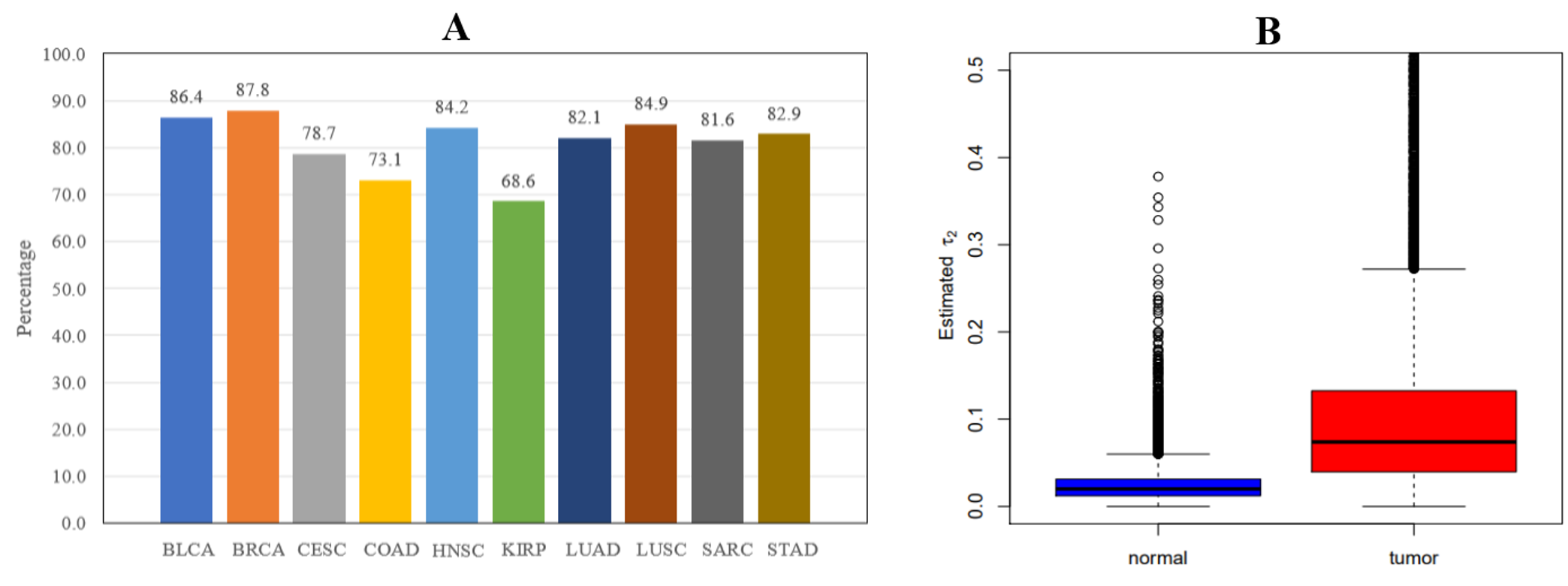

Supplement: S5 Fig — (A) Proportion of overlapped methylation-regulated genes discovered in various cancer tumor tissues and these discovered in normal tissue. (B) Estimated values of τ2in the BRCA tissue and in the normal tissue. Here, τ2 can be employed to quantify the magnitude of the methylation effect on expression, the sample size for the normal tissue is combined and analyzed across all the 10 cancers. (TIF) [file pcbi.1009250.s005.tif]

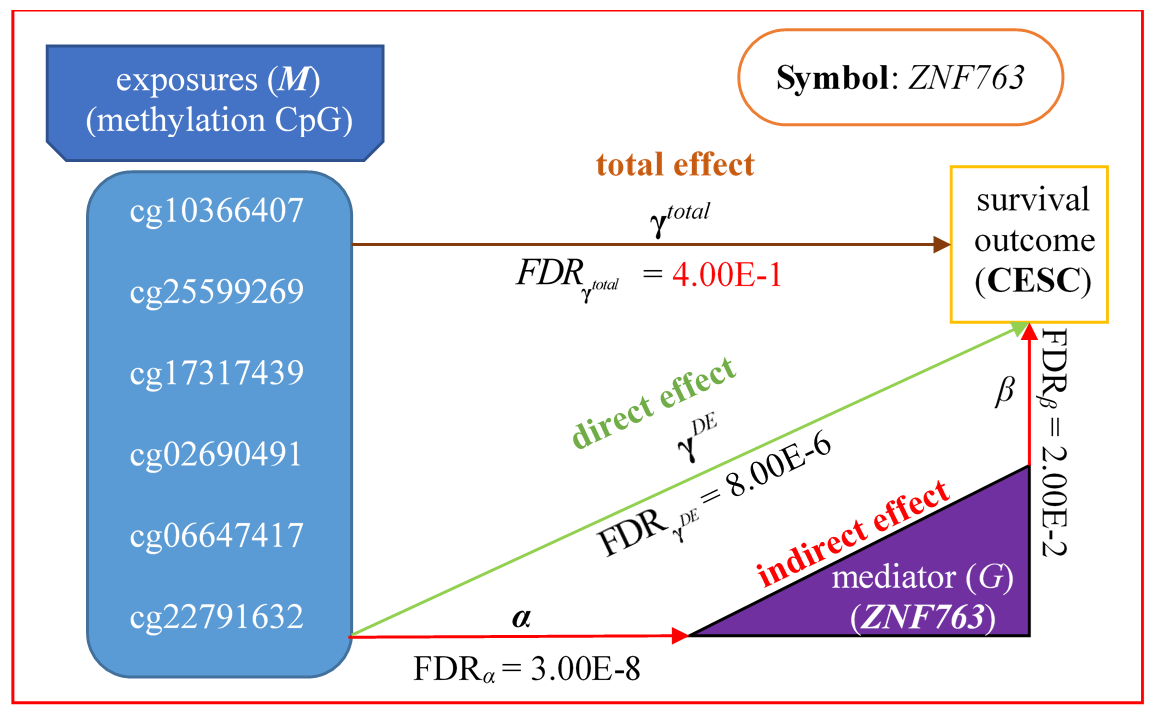

Supplement: S6 Fig — Please refer to Fig 1 for the interpretation of these parameters shown herein. (TIF) [file pcbi.1009250.s006.tif]

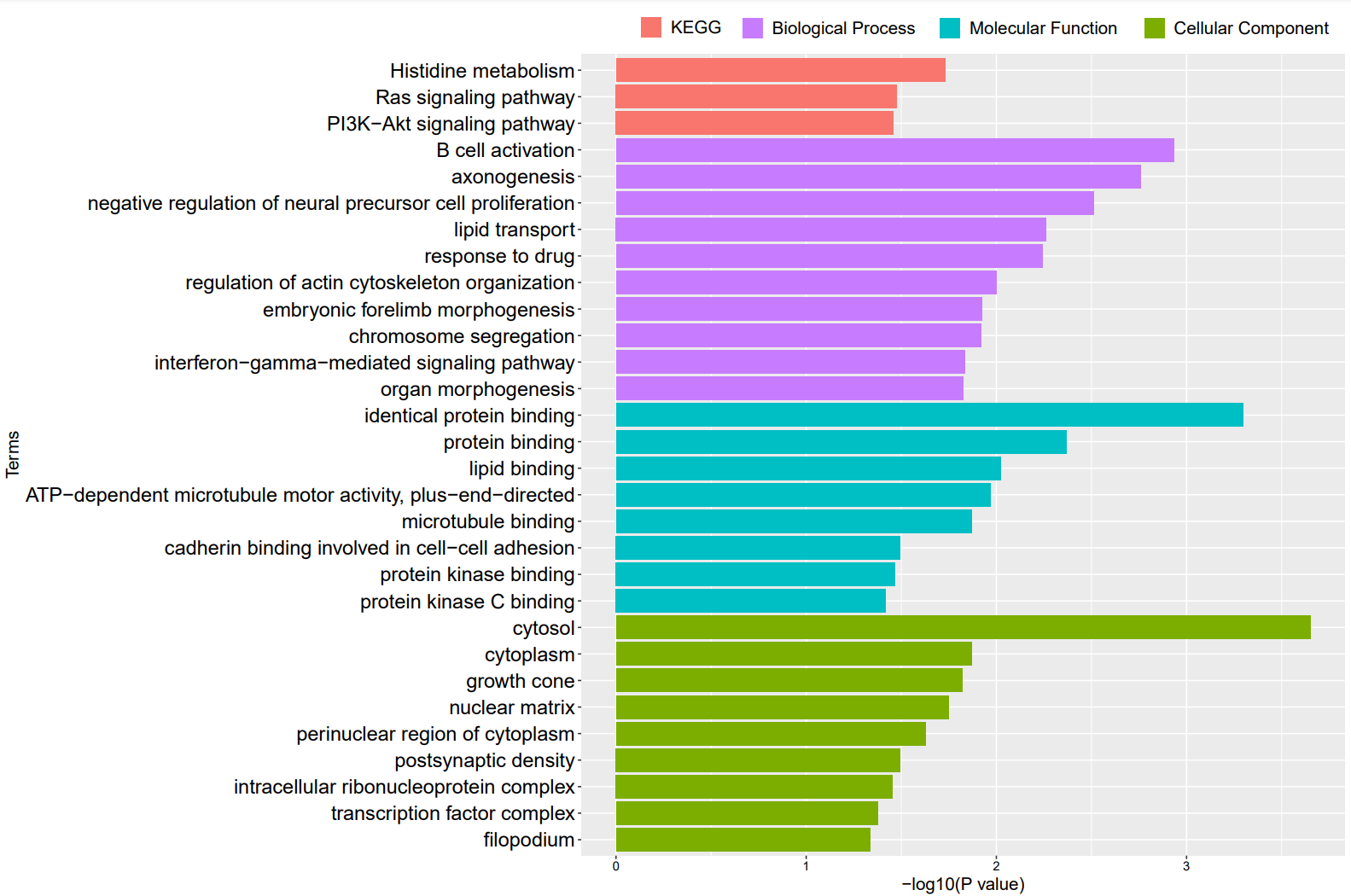

Supplement: S7 Fig — (TIF) [file pcbi.1009250.s007.tif]

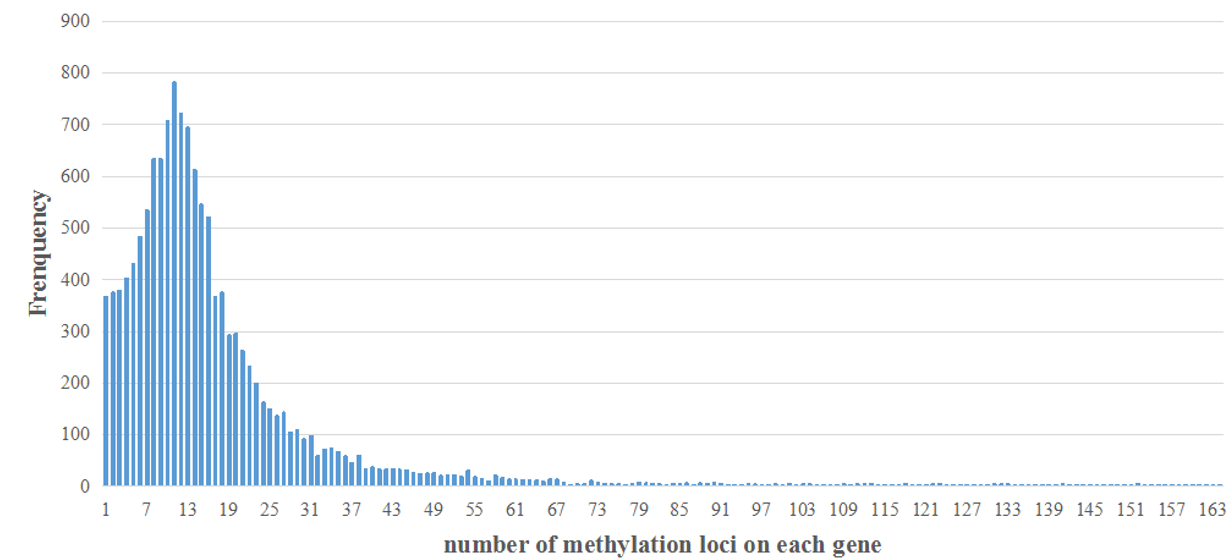

Supplement: S8 Fig — (TIF) [file pcbi.1009250.s008.tif]

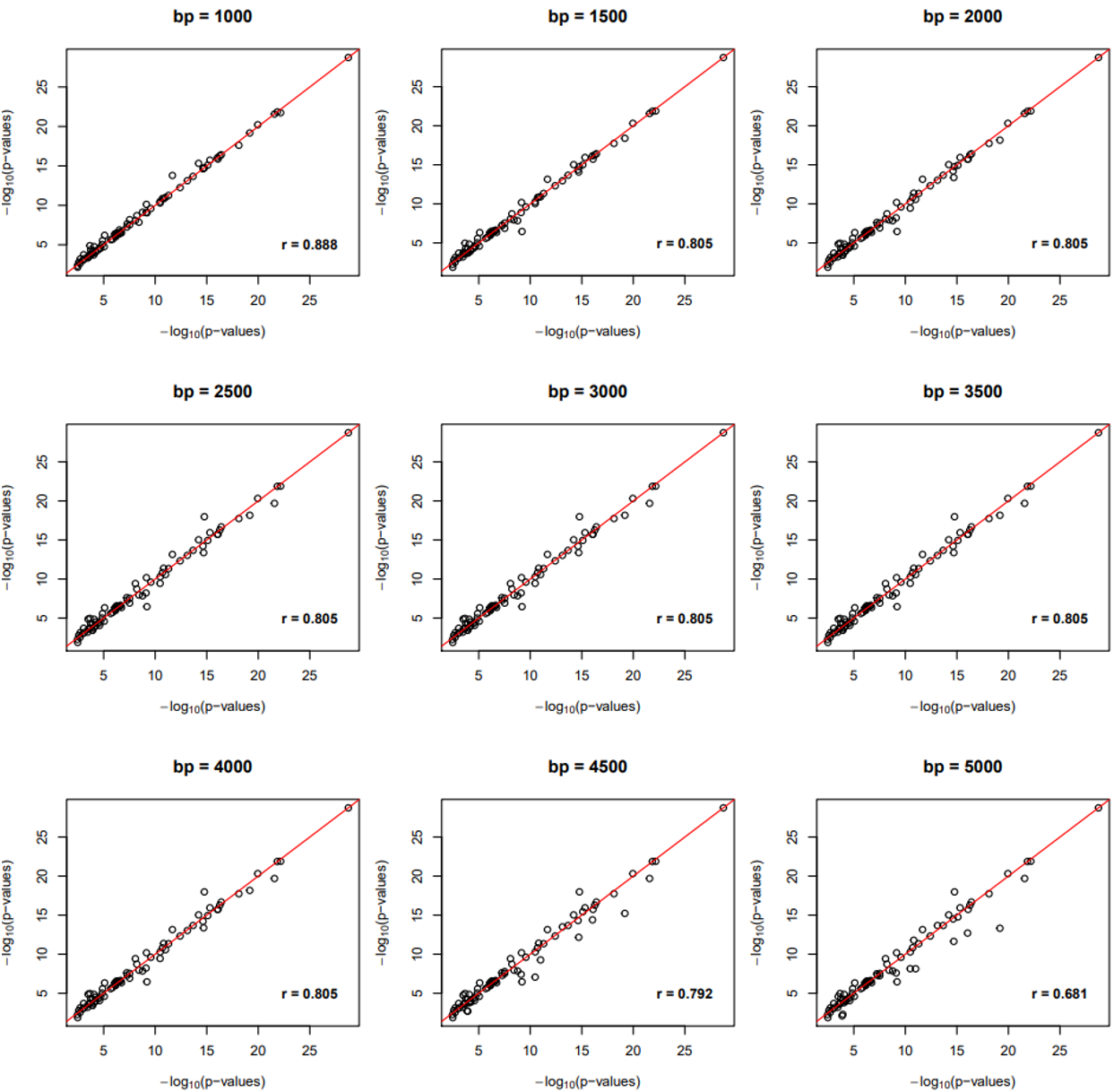

Supplement: S9 Fig — Here, various distances before the TSS were extended, ranging from 500bp to 5000bp with an increment of 500bp. For each extension, the methylations within that extended region and gene body were included to examine their relationship with gene expression using the variance-component score test. The P-values calculated with methylations within a 500bp upstream of the TSS were treated as the reference. (TIF) [file pcbi.1009250.s009.tif]
