# Supplementary material for: IUSMMT: Survival mediation analysis of gene expression with multiple DNA methylation exposures and its application to cancers of TCGA
Source: PLoS Comput Biol. 2021 Aug 31;17(8):e1009250. doi: 10.1371/journal.pcbi.1009250 (PMC8437300; doi:10.1371/journal.pcbi.1009250)
Supplement: S1 Table — (DOCX) [file pcbi.1009250.s010.docx]

**S1 Table**. True and estimated proportion parameters (mean and standard deviation) in the three-component mixture null distribution under the five simulation scenarios and different sample sizes and different numbers of mediators

| **Dense null** | ***κ*_00_ = 0.1** | ***κ*_01_ = 0.15** | ***κ*_10_ = 0.75** | ***κ*_11_ = 0** |
| --- | --- | --- | --- | --- |
| n = 250 | 0.550 (0.185) | 0.099 (0.030) | 0.351 (0.182) | 0.000 (0.000) |
| n = 400 | 0.423 (0.190) | 0.123 (0.026) | 0.454 (0.188) | 0.000 (0.000) |
| n = 548 | 0.349 (0.180) | 0.136 (0.020) | 0.515 (0.178) | 0.000 (0.000) |
|  | ***κ*_00_ = 0.1** | ***κ*_01_ = 0.3** | ***κ*_10_ = 0.6** | ***κ*_11_ = 0** |
| n = 250 | 0.527 (0.160) | 0.192 (0.062) | 0.281 (0.146) | 0.000 (0.000) |
| n = 400 | 0.395 (0.160) | 0.242 (0.053) | 0.364 (0.151) | 0.000 (0.000) |
| n = 548 | 0.321 (0.149) | 0.268 (0.040) | 0.411 (0.143) | 0.000 (0.000) |
|  | ***κ*_00_ = 0.1** | ***κ*_01_ = 0.35** | ***κ*_10_ = 0.55** | ***κ*_11_ = 0** |
| n = 250 | 0.520 (0.152) | 0.223 (0.071) | 0.256 (0.134) | 0.000 (0.000) |
| n = 400 | 0.385 (0.152) | 0.281 (0.062) | 0.334 (0.138) | 0.000 (0.000) |
| n = 548 | 0.312 (0.139) | 0.312 (0.047) | 0.377 (0.131) | 0.000 (0.000) |
|  | ***κ*_00_ = 0.1** | ***κ*_01_ = 0.65** | ***κ*_10_ = 0.25** | ***κ*_11_ = 0** |
| n = 250 | 0.473 (0.147) | 0.410 (0.133) | 0.116 (0.061) | 0.000 (0.000) |
| n = 400 | 0.329 (0.133) | 0.518 (0.117) | 0.152 (0.063) | 0.000 (0.000) |
| n = 548 | 0.253 (0.106) | 0.575 (0.088) | 0.172 (0.060) | 0.000 (0.000) |
|  | ***κ*_00_ = 0.1** | ***κ*_01_ = 0.7** | ***κ*_10_ = 0.2** | ***κ*_11_ = 0** |
| n = 250 | 0.465 (0.152) | 0.442 (0.143) | 0.093 (0.049) | 0.000 (0.000) |
| n = 400 | 0.320 (0.135) | 0.558 (0.126) | 0.122 (0.050) | 0.000 (0.000) |
| n = 548 | 0.244 (0.107) | 0.618 (0.095) | 0.138 (0.047) | 0.000 (0.000) |
|  | ***κ*_00_ = 0.1** | ***κ*_01_ = 0.85** | ***κ*_10_ = 0.05** | ***κ*_11_ = 0** |
| n = 250 | 0.441 (0.175) | 0.536 (0.174) | 0.022 (0.012) | 0.001 (0.001) |
| n = 400 | 0.293 (0.155) | 0.675 (0.154) | 0.031 (0.012) | 0.001 (0.001) |
| n = 548 | 0.215 (0.116) | 0.750 (0.116) | 0.034 (0.012) | 0.000 (0.002) |
| **Sparse null** | ***κ*_00_ = 0.9** | ***κ*_01_ = 0** | ***κ*_10_ = 0.1** | ***κ*_11_ = 0** |
| n = 250 | 0.917 (0.028) | 0.001 (0.002) | 0.083 (0.028) | 0.000 (0.000) |
| n = 400 | 0.913 (0.023) | 0.001 (0.002) | 0.086 (0.024) | 0.000 (0.000) |
| n = 548 | 0.902 (0.024) | 0.001 (0.002) | 0.097 (0.025) | 0.000 (0.000) |
|  | ***κ*_00_ = 0.9** | ***κ*_01_ = 0.1** | ***κ*_10_ = 0** | ***κ*_11_ = 0** |
| n = 250 | 0.899 (0.021) | 0.100 (0.021) | 0.000 (0.001) | 0.000 (0.000) |
| n = 400 | 0.899 (0.013) | 0.101 (0.013) | 0.000 (0.001) | 0.000 (0.000) |
| n = 548 | 0.891 (0.019) | 0.109 (0.019) | 0.000 (0.001) | 0.000 (0.000) |
|  | ***κ*_00_ = 0.95** | ***κ*_01_ = 0** | ***κ*_10_ = 0.05** | ***κ*_11_ = 0** |
| n = 250 | 0.954 (0.020) | 0.001 (0.002) | 0.045 (0.021) | 0.000 (0.000) |
| n = 400 | 0.953 (0.013) | 0.002 (0.006) | 0.045 (0.015) | 0.000 (0.001) |
| n = 548 | 0.945 (0.019) | 0.001 (0.002) | 0.054 (0.019) | 0.000 (0.000) |
|  | ***κ*_00_ = 0.95** | ***κ*_01_ = 0.05** | ***κ*_10_ = 0** | ***κ*_11_ = 0** |
| n = 250 | 0.941 (0.016) | 0.059 (0.016) | 0.000 (0.001) | 0.000 (0.000) |
| n = 400 | 0.945 (0.011) | 0.054 (0.011) | 0.000 (0.001) | 0.000 (0.000) |
| n = 548 | 0.938 (0.018) | 0.062 (0.018) | 0.000 (0.001) | 0.000 (0.000) |
|  | ***κ*_00_ = 0.99** | ***κ*_01_ = 0** | ***κ*_10_ = 0.01** | ***κ*_11_ = 0** |
| n = 250 | 0.987 (0.015) | 0.004 (0.010) | 0.008 (0.014) | 0.000 (0.000) |
| n = 400 | 0.994 (0.008) | 0.003 (0.006) | 0.003 (0.006) | 0.000 (0.000) |
| n = 548 | 0.985 (0.020) | 0.005 (0.014) | 0.010 (0.018) | 0.000 (0.000) |
|  | ***κ*_00_ = 0.99** | ***κ*_01_ = 0.01** | ***κ*_10_ = 0** | ***κ*_11_ = 0** |
| n = 250 | 0.977 (0.017) | 0.022 (0.017) | 0.000 (0.001) | 0.001 (0.003) |
| n = 400 | 0.986 (0.011) | 0.013 (0.011) | 0.000 (0.001) | 0.001 (0.001) |
| n = 548 | 0.977 (0.018) | 0.023 (0.019) | 0.000 (0.001) | 0.000 (0.000) |
| **Complete null** | ***κ*_00_ = 1** | ***κ*_01_ = 0** | ***κ*_10_ = 0** | ***κ*_11_ = 0** |
| n = 250 | 0.993 (0.011) | 0.005 (0.010) | 0.002 (0.006) | 0.000 (0.000) |
| n = 400 | 0.998 (0.003) | 0.001 (0.003) | 0.001 (0.002) | 0.000 (0.000) |
| n = 548 | 0.994 (0.013) | 0.004 (0.012) | 0.003 (0.008) | 0.000 (0.000) |
| **Sparse alternative (A)** | ***κ*_00_ = 0.9** | ***κ*_01_ = 0** | ***κ*_10_ = 0** | ***κ*_11_ = 0.1** |
| n = 250 | 0.900 (0.021) | 0.055 (0.020) | 0.033 (0.018) | 0.013 (0.018) |
| n = 400 | 0.892 (0.017) | 0.047 (0.026) | 0.025 (0.018) | 0.036 (0.028) |
| n = 548 | 0.870 (0.029) | 0.061 (0.031) | 0.040 (0.026) | 0.028 (0.028) |
| **(B)** | ***κ*_00_ = 0.95** | ***κ*_01_ = 0** | ***κ*_10_ = 0** | ***κ*_11_ = 0.05** |
| n = 250 | 0.947 (0.014) | 0.031 (0.011) | 0.018 (0.011) | 0.004 (0.008) |
| n = 400 | 0.940 (0.012) | 0.030 (0.015) | 0.017 (0.011) | 0.013 (0.013) |
| n = 548 | 0.930 (0.015) | 0.035 (0.015) | 0.024 (0.014) | 0.010 (0.013) |
| **(C)** | ***κ*_00_ = 0.99** | ***κ*_01_ = 0** | ***κ*_10_ = 0** | ***κ*_11_ = 0.01** |
| n = 250 | 0.978 (0.014) | 0.018 (0.016) | 0.003 (0.005) | 0.000 (0.000) |
| n = 400 | 0.986 (0.009) | 0.012 (0.010) | 0.002 (0.004) | 0.000 (0.001) |
| n = 548 | 0.980 (0.015) | 0.017 (0.016) | 0.003 (0.005) | 0.000 (0.000) |
| **Dense alternative (D)** | ***κ*_00_ = 0.1** | ***κ*_01_ = 0.05** | ***κ*_10_ = 0.75** | ***κ*_11_ = 0.1** |
| n = 250 | 0.501 (0.210) | 0.100 (0.030) | 0.399 (0.206) | 0.000 (0.000) |
| n = 400 | 0.364 (0.210) | 0.119 (0.025) | 0.513 (0.208) | 0.004 (0.012) |
| n = 548 | 0.292 (0.191) | 0.125 (0.028) | 0.572 (0.189) | 0.011 (0.024) |
| **(E)** | ***κ*_00_ = 0.1** | ***κ*_01_ = 0.2** | ***κ*_10_ = 0.6** | ***κ*_11_ = 0.1** |
| n = 250 | 0.478 (0.182) | 0.193 (0.062) | 0.329 (0.170) | 0.000 (0.000) |
| n = 400 | 0.337 (0.179) | 0.238 (0.051) | 0.421 (0.171) | 0.004 (0.012) |
| n = 548 | 0.262 (0.161) | 0.258 (0.040) | 0.470 (0.157) | 0.010 (0.024) |
| **(F)** | ***κ*_00_ = 0.1** | ***κ*_01_ = 0.25** | ***κ*_10_ = 0.55** | ***κ*_11_ = 0.1** |
| n = 250 | 0.472 (0.175) | 0.224 (0.072) | 0.304 (0.158) | 0.000 (0.000) |
| n = 400 | 0.329 (0.170) | 0.277 (0.059) | 0.390 (0.159) | 0.004 (0.012) |
| n = 548 | 0.252 (0.151) | 0.301 (0.045) | 0.436 (0.145) | 0.011 (0.024) |
| **(G)** | ***κ*_00_ = 0.1** | ***κ*_01_ = 0.55** | ***κ*_10_ = 0.25** | ***κ*_11_ = 0.1** |
| n = 250 | 0.425 (0.159) | 0.411 (0.133) | 0.164 (0.085) | 0.000 (0.000) |
| n = 400 | 0.273 (0.139) | 0.513 (0.112) | 0.208 (0.083) | 0.006 (0.016) |
| n = 548 | 0.200 (0.104) | 0.558 (0.079) | 0.225 (0.072) | 0.017 (0.028) |
| **(H)** | ***κ*_00_ = 0.1** | ***κ*_01_ = 0.6** | ***κ*_10_ = 0.2** | ***κ*_11_ = 0.1** |
| n = 250 | 0.417 (0.162) | 0.443 (0.144) | 0.140 (0.073) | 0.000 (0.000) |
| n = 400 | 0.265 (0.138) | 0.552 (0.120) | 0.177 (0.070) | 0.007 (0.017) |
| n = 548 | 0.193 (0.100) | 0.600 (0.085) | 0.189 (0.061) | 0.018 (0.029) |
| **(I)** | ***κ*_00_ = 0.1** | ***κ*_01_ = 0.75** | ***κ*_10_ = 0.05** | ***κ*_11_ = 0.1** |
| n = 250 | 0.394 (0.179) | 0.537 (0.174) | 0.070 (0.036) | 0.000 (0.000) |
| n = 400 | 0.241 (0.145) | 0.667 (0.143) | 0.081 (0.035) | 0.011 (0.022) |
| n = 548 | 0.172 (0.098) | 0.725 (0.097) | 0.077 (0.037) | 0.027 (0.034) |

Note: *κ*_10_ stands for the probability that the exposures are related to the mediator in the exposure-mediator model but the mediator is not associated with the survival outcome in the mediator-outcome model; *κ*_01_ stands for the probability that the exposures are not related to the mediator in the exposure-mediator model but the mediator is associated with the survival outcome in the mediator-outcome model; *κ*_00_ stands for the probability that the exposures are not related to the mediator in the exposure-mediator model and the mediator is not associated with the survival outcome in the mediator-outcome model; *κ*_11_ stands for the probability of the existence of mediation effects; for the nine cases in the alternative (including sparse and dense alternative), for simplicity we use A-I to represent them. The number of genes (i.e., mediators) was here set to 10^4^.
