# Supplementary material for: IUSMMT: Survival mediation analysis of gene expression with multiple DNA methylation exposures and its application to cancers of TCGA
Source: PLoS Comput Biol. 2021 Aug 31;17(8):e1009250. doi: 10.1371/journal.pcbi.1009250 (PMC8437300; doi:10.1371/journal.pcbi.1009250)
Supplement: S2 Table — (DOCX) [file pcbi.1009250.s011.docx]

**S2 Table**. Comparison of the influence of different numbers of mediators on the bias of the estimators for these proportion parameters

| **S=10^3^** | ***κ*_00_ = 0.1** | ***κ*_01_ = 0.75** | ***κ*_10_ = 0.05** | ***κ*_11_ = 0.1** |
| --- | --- | --- | --- | --- |
| **n = 250** | 0.498 (0.214) | 0.401 (0.210) | 0.101 (0.032) | 0.000 (0.001) |
| **n = 400** | 0.358 (0.210) | 0.515 (0.208) | 0.120 (0.029) | 0.007 (0.019) |
| **n = 548** | 0.282 (0.204) | 0.580 (0.203) | 0.127 (0.037) | 0.012 (0.032) |
| **S=10^4^** | ***κ*_00_ = 0.1** | ***κ*_01_ = 0.75** | ***κ*_10_ = 0.05** | ***κ*_11_ = 0.1** |
| **n = 250** | 0.394 (0.179) | 0.536 (0.174) | 0.069 (0.037) | 0.000 (0.000) |
| **n = 400** | 0.241 (0.145) | 0.667 (0.143) | 0.081 (0.035) | 0.011 (0.021) |
| **n = 548** | 0.172 (0.098) | 0.725 (0.098) | 0.076 (0.036) | 0.026 (0.034) |

Note: to evaluate the impact of various numbers of mediators on the bias of the estimators, we implemented an additional simulation with 10^3^ genes with *κ*_11_ = 0.10, *κ*_10_ = 0.75, *κ*_01_ = 0.05, and *κ*_00_ = 0.10 (close to proportions obtained from our real applications).
