# Supplementary material for: IUSMMT: Survival mediation analysis of gene expression with multiple DNA methylation exposures and its application to cancers of TCGA
Source: PLoS Comput Biol. 2021 Aug 31;17(8):e1009250. doi: 10.1371/journal.pcbi.1009250 (PMC8437300; doi:10.1371/journal.pcbi.1009250)
Supplement: S3 Table — (DOCX) [file pcbi.1009250.s012.docx]

**S3 Table**. Data process of the ten TCGA cancers used in our mediation analysis

| **Cancers** | **full name** | **initial data** | | | **after quality control** | | |
| --- | --- | --- | --- | --- | --- | --- | --- |
|  |  | ***n*_1_** | ***n*_2_** | ***n*_3_** | ***n*** | ***m*_1_** | ***m*_2_** |
| **BLCA** | Bladder urothelial carcinoma | 434 | 426 | 400 | 317 | 395896 | 14598 |
| **BRCA** | Breast invasive carcinoma | 888 | 1218 | 1060 | 548 | 395611 | 14769 |
| **CESC** | Cervical squamous cell carcinoma and endocervical adenocarcinoma | 312 | 308 | 287 | 197 | 395456 | 14678 |
| **COAD** | Colon adenocarcinoma | 337 | 329 | 270 | 180 | 395507 | 14596 |
| **HNSC** | Head and Neck squamous cell carcinoma | 580 | 566 | 440 | 374 | 395801 | 14800 |
| **KIRP** | Kidney renal papillary cell carcinoma | 321 | 323 | 256 | 176 | 395806 | 14498 |
| **LUAD** | Lung adenocarcinoma | 492 | 576 | 486 | 345 | 395642 | 14788 |
| **LUSC** | Lung squamous cell carcinoma | 415 | 553 | 481 | 269 | 395860 | 15019 |
| **SARC** | Sarcoma | 269 | 265 | 258 | 226 | 395439 | 14500 |
| **STAD** | Stomach adenocarcinoma | 398 | 450 | 379 | 226 | 395660 | 14978 |

Note: *n*_1_, *n*_2_ and *n*_3_ indicate the sample size of methylation CpG site, gene expression and clinical datasets, respectively; *n* is the number of sample size shared by the three types of datasets after quality control; *m*_1_ and *m*_2_ denote the number of methylation loci and gene expressions after quality control, respectively.
