# Supplementary material for: IUSMMT: Survival mediation analysis of gene expression with multiple DNA methylation exposures and its application to cancers of TCGA
Source: PLoS Comput Biol. 2021 Aug 31;17(8):e1009250. doi: 10.1371/journal.pcbi.1009250 (PMC8437300; doi:10.1371/journal.pcbi.1009250)
Supplement: S4 Table — (DOCX) [file pcbi.1009250.s013.docx]

**S4 Table**. Basic characteristics of the ten TCGA cancer datasets

| **Cancer** | ***n*** | **G-M pairs** | **age** | **gender** | **event (%)** | **median survival time** | | |
| --- | --- | --- | --- | --- | --- | --- | --- | --- |
|  |  |  |  |  |  | **all** | **event** | **censor** |
| **BLCA** | 317 | 13125 | 69.0±10.0 | 237/80 | 147 (46.4) | 18.3 | 13.8 | 24.3 |
| **BRCA** | 548 | 13270 | 59.0±13.0 | 0/548 | 73 (13.3) | 29.6 | 39.1 | 28.4 |
| **CESC** | 197 | 13202 | 48.1±13.8 | 0/197 | 47 (23.9) | 22.9 | 21.3 | 24.1 |
| **COAD** | 180 | 13132 | 66.2±12.9 | 98/82 | 45 (25.0) | 23.6 | 15.7 | 24.2 |
| **HNSC** | 374 | 13317 | 61.1±12.4 | 267/107 | 157 (42.0) | 22.6 | 14.5 | 29.2 |
| **KIRP** | 176 | 13029 | 63.0±11.5 | 132/44 | 27 (15.3) | 25.7 | 19.9 | 25.9 |
| **LUAD** | 345 | 13293 | 65.7±10.0 | 155/190 | 127 (36.8) | 20.8 | 20.2 | 20.9 |
| **LUSC** | 269 | 13487 | 67.4±8.9 | 198/71 | 109 (40.5) | 20.3 | 18.1 | 21.3 |
| **SARC** | 226 | 13035 | 60.8±14.7 | 114/112 | 85 (37.6) | 34.9 | 24.6 | 36.9 |
| **STAD** | 226 | 13457 | 64.9±9.9 | 141/85 | 86 (38.1) | 16.5 | 13.1 | 19.4 |

Note: G-M pairs: the number of gene-methylation pairs; all these descriptions are undertaken after the gene-methylation match and quality control.
