# Supplementary material for: IUSMMT: Survival mediation analysis of gene expression with multiple DNA methylation exposures and its application to cancers of TCGA
Source: PLoS Comput Biol. 2021 Aug 31;17(8):e1009250. doi: 10.1371/journal.pcbi.1009250 (PMC8437300; doi:10.1371/journal.pcbi.1009250)
Supplement: S5 Table — (DOCX) [file pcbi.1009250.s014.docx]

**S5 Table**. Results for testing for the proportional hazards assumption in the used Cox model

| **Cancer** | *P* |
| --- | --- |
| **BLCA** | 0.622 |
| **BRCA** | 0.050 |
| **CESC** | 0.577 |
| **COAD** | 0.079 |
| **HNSC** | 0.845 |
| **KIRP** | 0.057 |
| **LUAD** | 0.253 |
| **LUSC** | 0.346 |
| **SARC** | 0.793 |
| **STAD** | 0.868 |

Note: The test for the proportional hazards assumption was conducted with the weighted residual method proposed in [1] by using the cox.zph function in the survival package. For each cancer, only covariates were considered in the Cox model when conducting the test.

### References

1. Lutz SM, Fingerlin TE, Hokanson JE, Lange C (2017) A general approach to testing for pleiotropy with rare and common variants. Genet Epidemiol 41: 163-170.
