# Supplementary material for: IUSMMT: Survival mediation analysis of gene expression with multiple DNA methylation exposures and its application to cancers of TCGA
Source: PLoS Comput Biol. 2021 Aug 31;17(8):e1009250. doi: 10.1371/journal.pcbi.1009250 (PMC8437300; doi:10.1371/journal.pcbi.1009250)
Supplement: S1 Text — (DOCX) [file pcbi.1009250.s015.docx]

### S1 Text. Effects in survival mediation analysis with multiple exposures, and identifiability assumptions

***Natural direct effect and natural indirect effect***

Let $T_{m_{k}}$ denote the counterfactual event time if *M_k_* is set to *m_k_*; likewise, let $T_{m_{k}g}$ denote the counterfactual event time if *M_k_* is set to *m_k_* and *G* is set to *g*. Let $G_{m_{k}}$ be the counterfactual value of the mediator if *M_k_* is set to *m_k_;* and ***X*** be the covariates. Under the linear regression model (Equation 2 in the main text) for the exposure-mediator relationship and the Cox proportional hazards model (Equation 4 in the main text) for the mediator-outcome relationship, let

$$h_{T_{_{T_{m_{k}G_{m_{k}^{*}}}}}}(t|m_{k}, G_{m_{k}^{*}}, \mathbf{X}) = \frac{f_{T_{m_{k}G_{m_{k}^{*}}}}(t|m_{k}, G_{m_{k}^{*}}, \mathbf{X})}{S_{T_{m_{k}G_{m_{k}^{*}}}}(t|m_{k}, G_{m_{k}^{*}}, \mathbf{X})}$$

where $f_{T_{m_{k}G_{m_{k}^{*}}}}(t|m_{k}, G_{m_{k}^{*}}, \mathbf{X})$ and $S_{T_{m_{k}G_{m_{k}^{*}}}}(t|m_{k}, G_{m_{k}^{*}}, \mathbf{X})$ denote the conditional density and survival functions for $T_{m_{k}G_{m_{k}^{*}}}$, respectively. We have

$$h_{T_{_{T_{m_{k}G_{m_{k}^{*}}}}}}(t|m_{k}, G_{m_{k}^{*}}, X) = h_{T}(t|0, 0, 0)\exp(\sum_{k = 1}^{K} (m_{k}\text{γ}_{k}^{DE}) + \mathbf{X}w_{3})r(t; m_{k}, m_{k}^{*}, \mathbf{X})$$

where $\text{γ}_{k}^{DE}$ is the direct effect of the *k*^th^ methylation CpG site, ***w***_3_ = (*w*_31, …_, *w*_3_*_L_*) denotes the vector of the effect sizes of *L* covariates in the mediator-outcome model, and

$$r(t; m_{k}, m_{k}^{*}, \mathbf{X}) = \frac{\int e^{\beta g}\exp(-\Lambda_{T}(t|0, 0, 0)e^{\sum_{k = 1}^{K} (m_{k}\text{γ}_{k}^{DE}) + g\beta+ \mathbf{X}w_{3}})dP_{G}(g|m_{k}^{*}, \mathbf{X})}{\int\exp(-\Lambda_{T}(t|0, 0, 0)e^{\sum_{k = 1}^{K} (m_{k}\text{γ}_{k}^{DE}) + g\beta+ \mathbf{X}w_{3}})dP_{G}(g|m_{k}^{*}, \mathbf{X})}$$

where *β* is the effect size of gene expression. Since *G* is assumed to be normally distributed, we have

$\begin{matrix} r(t; m_{k}, m_{k}^{*}, \mathbf{X}) = e^{\beta(\sum_{k = 1}^{K} (m_{m}^{*}\alpha_{k}) + \mathbf{Xw}_{2}) + \frac{1}{2}\beta^{2}\text{σ}^{2}} \\ \times\frac{\int\exp(-\Lambda_{T}(t|0, 0, 0)e^{\beta^{2} + \sum_{k = 1}^{K} (m_{k}\text{γ}_{k}^{DE}) + g\beta+ \mathbf{X}w_{3}})\exp\left(-\frac{(g - \sum_{k = 1}^{K} (m_{m}^{*}\alpha_{k}))^{2}}{2}\right)dg}{\int\exp(\text{-}\Lambda_{T}(t|0, 0, 0)e^{\sum_{k = 1}^{K} (m_{k}\text{γ}_{k}^{DE}) + g\beta+ \mathbf{X}w_{3}})\exp\left(-\frac{(g - \sum_{k = 1}^{K} (m_{m}^{*}\alpha_{k}))^{2}}{2}\right)dg} \end{matrix}$ where σ^2^ is the variance of the mediator in the exposure-mediator model; *α_k_* is the effect size of the *k*^th^ methylation CpG site on the gene expression; ***w***_2_ = (*w*_21, …_, *w*_2_*_L_*) denotes the vector of the effect sizes of *L* covariates in the exposure-mediator model. The equation can be approximated by

$$\exp(\beta(\sum_{k = 1}^{K} (m_{m}^{*}\alpha_{k}) + \mathbf{Xw}_{2}) + \frac{1}{2}\beta^{2}\text{σ}^{2})$$

if $\Lambda_{T}(t|0, 0, 0)$ is small. Thus, finally, we have the following approximation

$$\begin{matrix} \log\left\{h_{T_{m_{k}G_{m_{k}^{*}}}}(t|m_{k}, G_{m_{k}^{*}}, \mathbf{X})\right\} = \log(h_{T}(t|0, 0, 0)) + \sum_{k = 1}^{K} (m_{k}\text{γ}_{k}^{DE}) + \mathbf{Xw}_{3} \\ + \beta(\sum_{k = 1}^{K} (\alpha_{k}m_{m}^{*}) + \mathbf{Xw}_{2}) + \frac{1}{2}\beta^{2}\text{σ}^{2} \end{matrix}$$

From this formula, under the assumption of absence of interaction between the exposures and the mediator (i.e., no interaction between DNA methylation CpG site and gene expression) [1,2], the natural direct effect (NDE) and the natural indirect effect (NIE) can then be expressed as

$$\begin{matrix} \log(\text{NDE}) = \log\left\{h_{T_{m_{k}G_{m_{k}}}}(t|m_{k}, G_{G_{m_{k}}}, X)\right\} - \log\left\{h_{T_{m_{k}G_{m_{k}^{*}}}}(t|m_{k}, G_{m_{k}^{*}}, X)\right\} \\ = \sum_{k=1}^{K} \text{γ}_{k}^{DE}(m_{k} - m_{k}^{*}) \\ \text{log(NIE)} \text{=} \log\left\{h_{T_{m_{k}G_{m_{k}}}}(t|m_{k}, G_{m_{k}}, X)\right\} - \log\left\{h_{T_{m_{k}G_{m_{k}^{*}}}}(t|m_{k}, G_{m_{k}^{*}}, X)\right\} \\ = \sum_{k=1}^{K} \beta\alpha_{k}(m_{k}-m_{k}^{*}) \end{matrix}$$

In particular, $\log(\text{NDE}) = \sum_{k=1}^{K} \text{γ}_{k}^{DE}$ and $\text{log(NIE)} = \sum_{k=1}^{K} \beta\alpha_{k}$ if $m_{k}-m_{k}^{*}$=1. In our main text, for each mediating gene we apply the sign of $\sum_{k=1}^{K} \text{γ}_{k}^{DE}$ and $\sum_{k=1}^{K} \beta\alpha_{k}$ to indicate the direction of NDE and NIE. We estimate $\text{γ}_{k}^{DE}$ (and τ_3_) using the EMMA algorithm [3], which is carried out via the EMMREML package [4]. We estimate $\alpha_{k}$ (and τ_2_) based on the Laplace approximation algorithm which is performed in the coxme package [5]

***Identifiability modeling assumptions in mediation analysis***

Furthermore, NDE and NIE can be interpreted in a causal way when identifiability assumptions are satisfied and individual mediation models are correctly constructed [6-8]. Besides the explicit assumption of temporal ordering between methylation, gene expression and survival outcome, these assumptions include: (i) the confounding between the methylation CpG site and the survival outcome must be correctly controlled; (ii) the confounding between the gene expression and the survival outcome must be correctly controlled; (iii) the confounding between the methylation CpG site and the gene expression must be correctly controlled; (iv) there should be no expression-survival confounders which are themselves affected by the methylation CpG site. The above assumptions are also known as sequential ignobility assumptions or no unmeasured confounding assumptions [9-13].

### References

1. VanderWeele TJ (2011) Causal mediation analysis with survival data. Epidemiology 22: 582-585.

2. Smith AA, Huang Y-T, Eliot M, Houseman EA, Marsit CJ, et al. (2014) A novel approach to the discovery of survival biomarkers in glioblastoma using a joint analysis of DNA methylation and gene expression. Epigenetics 9: 873-883.

3. Kang H, Zaitlen N, Wade C, Kirby A, Heckerman D, et al. (2008) Efficient control of population structure in model organism association mapping. Genetics 178: 1709 - 1723.

4. Akdemir D, Godfrey OU (2015) EMMREML: Fitting Mixed Models with Known Covariance Structures.

5. Therneau TM (2019) coxme: Mixed Effects Cox Models. R package version 2.2-14. <https://CRAN.R-project.org/package=coxme>.

6. VanderWeele TJ (2016) Mediation analysis: a practitioner's guide. Annu Rev Public Health 37: 17-32.

7. Robins JM, Greenland S (1992) Identifiability and exchangeability for direct and indirect effects. Epidemiology: 143-155.

8. MacKinnon DP, Fairchild AJ, Fritz MS (2007) Mediation analysis. Annu Rev Psychol 58: 593-614.

9. Imai K, Keele L, Tingley D (2010) A general approach to causal mediation analysis. Psychol Methods 15: 309.

10. Imai K, Keele L, Yamamoto T (2010) Identification, inference and sensitivity analysis for causal mediation effects. Stat Sci 25: 51-71.

11. Huang Y-T (2019) Genome-wide analyses of sparse mediation effects under composite null hypotheses. The Annals of Applied Statistics 13: 60-84.

12. Pearl J (2012) The causal mediation formula—a guide to the assessment of pathways and mechanisms. Prev Sci 13: 426-436.

13. Pearl J (2014) Interpretation and identification of causal mediation. Psychol Methods 19: 459-481.
