# Supplementary material for: IUSMMT: Survival mediation analysis of gene expression with multiple DNA methylation exposures and its application to cancers of TCGA
Source: PLoS Comput Biol. 2021 Aug 31;17(8):e1009250. doi: 10.1371/journal.pcbi.1009250 (PMC8437300; doi:10.1371/journal.pcbi.1009250)
Supplement: S2 Text — (DOCX) [file pcbi.1009250.s016.docx]

### S2 Text. Intersection-union test for the composite null hypothesis

Despite not as the commonly-used likelihood ratio test (LRT) as popular in statistical inference, the past few years have witnessed the increasing utilization of intersection-union test (IUT)in various statistical problems such as in bioequivalence testing [1], acceptance sampling problem [2] as well as contingency table problem [3,4]. Actually, it has been demonstrated under some certain cases that IUT is equivalent to LRT or more powerful compared to LRT [5], in which the alternative hypothesis is conveniently expressed as an intersection while the null hypothesis is expressed as a union [1,2,5-7].

Here we only present a basic introduction about IUT used for examining the significance of mediation effect; more theoretical details can be found in [1,2,5]. We first display the following three types for our mediation hypothesis testing problem

where Θ represents the parameter space and *H*_0_ is a composite hypothesis testing problem consisting of three sub-cases. Following the similar principle as IUT, the hypothesis testing for whether the mediation effect exists or not can be also expressed as

where ***φ*** = (***a***, *τ*_2_) and *S^c^* denotes the complementary set of *S*. It is easy to see that the test in is an IUT with finite sets. Note that, although there are three null hypotheses involved in IUT, no adjustment for multiplicity is needed because the overall null hypothesis *H*_0_ is rejected if and only if each of individual null hypotheses (e.g., *H*_01_, *H*_02_ and *H*_03_) (rather than any of individual null hypotheses; e.g., *H*_01_, *H*_02_ or *H*_03_) is rejected. According to the statement shown in [1,2,5], the following Theorem 1 ensures that IUT is a level-*α* test, which says that the type I error of IUT is guaranteed at most *α* once the rejection decision for *H*_0_ is made in terms of IUT.

**Theorem 1 for** **IUT**: If *R_i_* is a *level-α* rejection region for testing *H*_0_*_i_* versus *H*_1_*_i_* (*i* = 1, 2 and 3); then IUT with rejection region ** is a *level-α* test of *H*_0_ versus *H*_1_ given in .

Theorem 1 above illustrates that IUT can provide an overall type I error rate of *α* when examining the mediation effect without the need to adjust for multiplicity. However, IUT constructed via Theorem 1 may be quite conservative especially when the parameters are located near the boundary of the parameter space. The following theorem states when IUT is size-*α*.

**Theorem 2 for** **IUT**: For some *i* = 1, 2 or 3, suppose that *R_i_* is a *size-α* rejection region for testing *H*_0_*_i_* versus *H*_1_*_i_*; and suppose that *R_j_* is a *level-α* rejection region for testing *H*_0_*_j_* versus *H*_1_*_j_* for every *j*≠*i*. Further assume there exists a sequence of parameter points δ*_l_* such that

$$\lim_{l \to\infty}\mathrm{Prob}_{\delta_{l}}(R_{i}) = \alpha$$

and for every *j*≠*i*

$$\lim_{l \to\infty}\mathrm{Prob}_{\delta_{l}}(R_{j}) = 1$$

Then IUT with rejection region ** is a *size-α* test of *H*_0_ versus *H*_1_ given in .

Theorem 2 means under some regular conditions IUT will have size equal to *$\text{max}_{i}\left\{\alpha_{i}\right\}$*. However, Theorem 2 is every general and does not say how to achieve these conditions when implementing IUT. This motivates us to employ the use of the mixture null distribution (see below) to generate guaranteed *p*-value for IUT with the maximum *p*-value as statistic.

### References

1. Berger RL, Hsu JC (1996) Bioequivalence trials, intersection-union tests and equivalence confidence sets. Stat Sci 11: 283-319.

2. Berger RL (1982) Multiparameter Hypothesis Testing and Acceptance Sampling. Technometrics 24: 295-300.

3. Cohen A, Gatsonis C, Marden J (1983) Hypothesis tests and optimality properties in discrete multivariate analysis. In: Karlin S, Amemiya T, Goodman LA, editors. Studies in Econometrics, Time Series, and Multivariate Statistics: Academic Press. pp. 379-405.

4. Cohen A, Gatsonis C, Marden JI (1983) Hypothesis Testing for Marginal Probabilities in a 2 × 2 × 2 Contingency Table With Conditional Independence. J Am Stat Assoc 78: 920-929.

5. Berger RL (1997) Likelihood Ratio Tests and Intersection-Union Tests. In: Panchapakesan S, Balakrishnan N, editors. Advances in Statistical Decision Theory and Applications. Boston, MA: Birkhäuser Boston. pp. 225-237.

6. Berger RL (1989) Uniformly More Powerful Tests for Hypotheses concerning Linear Inequalities and Normal Means. J Am Stat Assoc 84: 192-199.

7. Sen PK, Tsai M-T (1999) Two-Stage Likelihood Ratio and Union–Intersection Tests for One-Sided Alternatives Multivariate Mean with Nuisance Dispersion Matrix. J Multivariate Anal 68: 264-282.
