# Supplementary material for: IUSMMT: Survival mediation analysis of gene expression with multiple DNA methylation exposures and its application to cancers of TCGA
Source: PLoS Comput Biol. 2021 Aug 31;17(8):e1009250. doi: 10.1371/journal.pcbi.1009250 (PMC8437300; doi:10.1371/journal.pcbi.1009250)
Supplement: S3 Text — (DOCX) [file pcbi.1009250.s017.docx]

### S3 Text. Three-component mixture null distribution

Suppose the *P*-value obtained from $H_{0}^{\alpha}$ ($H_{0}^{\alpha}$: ***α*** (or τ_2_) = 0) is *P****_α_*** and the *P*-value obtained from $H_{0}^{\beta}$ ($H_{0}^{\beta}$: *β* = 0) is *P_β_*. As done in IUT, we exploit *P*_max_ = max(*P****_α_***, *P_β_*) to evaluate the overall significance of the mediation effect but construct a three-component mixture null distribution rather than the general 0-1 uniform distribution for IUT. Specifically, for a given threshold value *u* for the significance evaluation, we have

$$\begin{matrix} \Pr(P_{\text{max,} j} \leq u|H_{0j}) = \Pr(P_{\text{max,} j} \leq u|H_{01, j})\Pr(H_{01, j}) \\ + \Pr(P_{\text{max,} j} \leq u|H_{10, j})\Pr(H_{10, j}) \\ + \Pr(P_{\text{max,} j} \leq u|H_{00, j})\Pr(H_{00, j}) \end{matrix}$$

which can be further expressed as

$$\begin{matrix} \Pr(P_{\text{max,} j} \leq u|H_{0j}) = \Pr(P_{a\text{,} j} \leq u|H_{01, j})\Pr(P_{\beta\text{,} j} \leq u|H_{01, j})\Pr(H_{01, j}) \\ + \Pr(P_{a\text{,} j} \leq u|H_{10, j})\Pr(P_{\beta\text{,} j} \leq u|H_{10, j})\Pr(H_{10, j}) \\ + \Pr(P_{a\text{,} j} \leq u|H_{00, j})\Pr(P_{\beta\text{,} j} \leq u|H_{00, j})\Pr(H_{00, j}) \end{matrix}$$

where $\Pr(P_{a\text{,} j} \leq u|H_{01, j})$ stands for the probability rejecting $H_{0}^{\alpha}$ under *H*_01_, under which the statistic for testing $H_{0}^{\alpha}$ theoretically follows a 0-1 uniform distribution; $\Pr(P_{\beta\text{,} j} \leq u|H_{01, j})$ is the power of rejecting $H_{0}^{\beta}$ under *H*_01_; similarly, $\Pr(P_{\beta\text{,} j} \leq u|H_{10, j})$ stands for the probability rejecting $H_{0}^{\beta}$ under *H*_10_, under which the statistic for testing $H_{0}^{\beta}$ theoretically follows a 0-1 uniform distribution; $(P_{a\text{,} j} \leq u|H_{10, j})$ is the power of rejecting $H_{0}^{\alpha}$ under *H*_10_; $\Pr(P_{a\text{,} j} \leq u|H_{00, j})$ and $\Pr(P_{\beta\text{,} j} \leq u|H_{00, j})$ are the probability rejecting $H_{0}^{\alpha}$ or $H_{0}^{\beta}$ under *H*_00_, under which both the test statistics theoretically follow a 0-1 uniform distribution. Notably, Equation holds because of the independence between ***α*** (τ_2_) and *β* under the sequential ignorability assumption [1-3]. Thus, we have

$$\begin{matrix} u = \Pr(P_{a\text{,} j} \leq u|H_{01, j}) \\ u = \Pr(P_{\beta\text{,} j} \leq u|H_{10, j}) \\ u = \Pr(P_{a\text{,} j} \leq u|H_{00, j}) \\ u = \Pr(P_{\beta\text{,} j} \leq u|H_{00, j}) \end{matrix}$$

In addition, let

$$\kappa_{01} = \Pr(H_{01}), \kappa_{10} = \Pr(H_{10}), \kappa_{00} = \Pr(H_{00})$$

and assume these proportions hold across all the mediation tests. Taking these together, we have the following expression for the proposed three-component mixture null distribution

$$\begin{matrix} \Pr(P_{\text{max}} \leq u|H_{0}) = \kappa_{10}p_{10}u + \kappa_{01}p_{01}u + \kappa_{00}u^{2} \\ p_{10} = \Pr(P_{a\text{,} j} \leq u|H_{10}) \\ p_{01} = \Pr(P_{\beta\text{,} j} \leq u|H_{01}) \end{matrix}$$

where *p*_10_ and *p*_01_ can be estimated via the Grenander method given in [4].

### References

1. Imai K, Keele L, Tingley D (2010) A general approach to causal mediation analysis. Psychol Methods 15: 309.

2. Imai K, Keele L, Yamamoto T (2010) Identification, inference and sensitivity analysis for causal mediation effects. Stat Sci 25: 51-71.

3. VanderWeele TJ (2016) Mediation analysis: a practitioner's guide. Annu Rev Public Health 37: 17-32.

4. Langaas M, Lindqvist BH, Ferkingstad E (2005) Estimating the proportion of true null hypotheses, with application to DNA microarray data. J R Stat Soc Ser B 67: 555-572.
