# Supplementary material for: IUSMMT: Survival mediation analysis of gene expression with multiple DNA methylation exposures and its application to cancers of TCGA
Source: PLoS Comput Biol. 2021 Aug 31;17(8):e1009250. doi: 10.1371/journal.pcbi.1009250 (PMC8437300; doi:10.1371/journal.pcbi.1009250)
Supplement: S4 Text — (DOCX) [file pcbi.1009250.s018.docx]

### S4 Text. Estimation of proportion parameters

The estimation of these proportion parameters required in can be easily implemented with methods that were well-established in the FDR literature [1-10]. Here, we estimate them with a simple non-parametric manner using in the calculation of q-values [6,8,9]. Let *κ*_0+_ be the null proportion of $P_{a\text{,} j}$ and *κ*_+0_ be the null proportion of $P_{\beta\text{,} j}$; then *κ*_0+_ and *κ*_+0_ can be conservatively estimated by

$$\begin{matrix} \hat{\kappa}_{0+}(\text{λ}_{a}) = \text{\#}\left\{P_{a\text{,} j} > \text{λ}_{a}\right\}\text{/(}m(1 - \text{λ}_{a})\text{)} \\ \hat{\kappa}_{+0}(\text{λ}_{\beta}) = \text{\#}\left\{P_{\beta\text{,} j} > \text{λ}_{\beta}\right\}\text{/(}m(1 - \text{λ}_{\beta})\text{)} \end{matrix}$$

where *m* is the total number of analyzed genes, and λ_α_ and λ_β_ are tuning parameters to determine whether an observed *P* value comes from the null case. In addition, *κ*_00_ is similarly estimated as

$$\hat{\kappa}_{00}\text{(λ}_{a}, \text{λ}_{\beta}) = \text{\#}\left\{P_{a\text{,} j} > \text{λ}_{a}, P_{\beta\text{,} j} > \text{λ}_{\beta}\right\}\text{/(}m(1 - \text{λ}_{a})(1 - \text{λ}_{\beta})\text{)}$$

It needs to highlight that a value of λ_α_ or λ_β_ that is much closer to one could lead to a higher confidence to guarantee the null of $P_{a\text{,} j}$ or $P_{\beta\text{,} j}$. However, a larger λ_α_ or λ_β_ would miss many $P_{a\text{,} j}$ and $P_{\beta\text{,} j}$ which actually come from the null distribution; in contrast, a smaller value of λ_α_ or λ_β_ that is closer to zero has the risk of incorporating non-null $P_{a\text{,} j}$ or $P_{\beta\text{,} j}$. Investigating the optimal value for λ_α_ or λ_β_ is beyond the scope of our study. In the present study, for simplicity we employ λ_α_ = λ_β_ = 0.5 following prior work [11]. Ultimately, it has

$$\begin{matrix} \hat{\kappa}_{01}(\text{λ}_{a}) = \hat{\kappa}_{0+}(\text{λ}_{a}) - \hat{\kappa}_{00}\text{(λ}_{a}, \text{λ}_{\beta}) \\ \hat{\kappa}_{10}(\text{λ}_{\beta}) = \hat{\kappa}_{+0}(\text{λ}_{\beta}) - \hat{\kappa}_{00}\text{(λ}_{a}, \text{λ}_{\beta}) \end{matrix}$$

Once the estimates of these proportions are obtained, the estimated mixture null distribution for *P*_max_ can be built to control FWER or FDR [11]. A comprehensive theoretical derivation with regards to the control of FWER or FDR can be conferred in [11]

### References

1. Jin J, Cai TT (2007) Estimating the Null and the Proportion of Nonnull Effects in Large-Scale Multiple Comparisons. J Am Stat Assoc 102: 495-506.

2. Jiang H, Doerge RW (2008) Estimating the proportion of true null hypotheses for multiple comparisons. Cancer Inform 6: 25-32.

3. Huang Y-T, Pan W-C (2016) Hypothesis test of mediation effect in causal mediation model with high-dimensional continuous mediators. Biometrics 72: 402-413.

4. Zhang H, Zheng Y, Zhang Z, Gao T, Joyce B, et al. (2016) Estimating and testing high-dimensional mediation effects in epigenetic studies. Bioinformatics 32: 3150-3154.

5. Efron B (2007) Size, power and false discovery rates. Ann Stat 35: 1351-1377.

6. Storey J (2003) The positive false discovery rate: a Bayesian interpretation and the q-value. Ann Stat 31: 2013 - 2035.

7. Efron B, Zhang NR (2011) False discovery rates and copy number variation. Biometrika 98: 251-271.

8. Storey J, Tibshirani R (2003) Statistical significance for genomewide studies. Proc Natl Acad Sci U S A 100: 9440 - 9445.

9. Storey J (2002) A direct approach to false discovery rates. J R Stat Soc Ser B 64: 479 - 498.

10. Efron B, Tibshirani R (2002) Empirical bayes methods and false discovery rates for microarrays. Genet Epidemiol 23: 70-86.

11. Dai JY, Stanford JL, LeBlanc M (2020) A Multiple-Testing Procedure for High-Dimensional Mediation Hypotheses. J Am Stat Assoc 10.1080/01621459.2020.1765785: 1-16.
