# Supplementary material for: IUSMMT: Survival mediation analysis of gene expression with multiple DNA methylation exposures and its application to cancers of TCGA
Source: PLoS Comput Biol. 2021 Aug 31;17(8):e1009250. doi: 10.1371/journal.pcbi.1009250 (PMC8437300; doi:10.1371/journal.pcbi.1009250)
Supplement: Attachment — Submitted filename: PCOMPBIOL-D-21-00050_Review_RNB.docx [file pcbi.1009250.s019.docx]

The authors have developed a novel gene-centric approach called IUSMMT (Intersection-Union Survival Mixture-adjusted Mediation Test) to delineate causal genes that mediate the effect of DNA methylation on cancer-specific survival. ISUMMT fits an empirical three-component mixture null distribution, and simulation studies demonstrated that it is higher powered than traditional approaches such as IUT (Intersection-Union Test) that rely on the uniform null distribution. The authors applied IUSMMT to 10 TCGA cancer types and revealed multiple genes (that were largely cancer-type specific) that exhibited mediation effects on cancer survival risk through the influence of altered DNA methylation at multiple CpG sites.

My overall impression is that the manuscript is a matter of interest since the new approach (IUSMMT) has potential utility in understanding the mechanism underlying the regulatory role of DNA methylation in cancer progression; and can aid in molecular classification as well as generate new insights into potential therapeutic targets. The analysis is clearly described, and the novel approach looks promising. However, I see some limitations that need addressing; and a few details that could be improved.

**Major revisions:**

1. The IUSMMT framework relies on the assumption that the methylation levels at CpG sites located within a gene can regulate the expression level in tumors, which in turn has an influence on patient survival. Certainly, noteworthy examples exist such as *BRCA1* promoter hypermethylation in ER- breast cancer. However, recent methylation studies in large cancer datasets (*Zhou et al.* 2018; *Meir et al.* 2020) have illustrated that a bulk of methylation changes observed in cancer are a consequence of global epigenetic remodelling machineries including i) global loss via replication related errors; ii) CpG island/ shore methylation gain associated with tumor proliferation. In such cases, altered expression of a handful of key genes would have an *in-trans* effect on DNA methylation at multiple (thousands) CpG sites over the cancer epigenome; and expression of the same key genes may also have a direct effect on survival.

However, under the mediation framework described in the manuscript, the above-mentioned trends would also result in an $alpha \neq0$ as well as $beta \neq0$ ($alpha$ and $beta$ are as defined in the manuscript). Following this result, one may incorrectly assume that this is an example of the expression of a gene the influence of DNA methylation on survival, when in fact, the gene expression may itself be driving the survival, and the altered DNA methylation are just passenger events. Since a large number of methylation loci are under the influence of such epigenetic machineries (particularly proliferative gain at CpG rich promoters, that are extensively covered by the hm450 array used for the 10 TCGA datasets), could the authors consider adding this state explicitly in the model to distinguish passenger methylation events from methylation events that are likely to have a direct/ or indirect (via expression mediation) influence on survival.

2. Could the authors elucidate the direction of the methylation alteration and gene expression change that is associated with worse survival? For example, how many were hypermethylation associated with repression?
3. Following from the previous 2 points, could the authors also indicate whether the genes that were induced (de-repressed) included proliferation or cell cycle related genes?
4. A large number of methylation-expression relationships observed in tumours are seen in normal tissues as well (but at a lower magnitude, e.g. field effects). Can the authors indicate how many of the expression-methylation relationships (identified under $alpha \neq0;$and identified under the IUSMMT) were also observed in TCGA normal tissues? The association with survival need not be investigated for normal tissues, so only comparisons in the expression-methylation relationship are requested.
5. In the gene-centric model, can the authors clarify how methylation loci were selected per gene? In the methods section under “Application to ten TCGA cancer datasets”, it is indicated that UCSC Xena was used for position annotation. Was this based on a standardised promoter definition or for does it span the entire gene? The authors make the assumption that *all DNA methylation CpG sites had an influence on the gene expression*, and as pointed out in the discussion, this may not hold realistically. Testing the sparse relationship may be out of the scope of the manuscript, and so in the absence of this, could the authors consider performing a sensitivity analysis to fine-tune the standardised definition of methylation loci selected for the genes (for example, identifying the most efficient distance to use around the promoter?).
6. Could the authors indicate whether distal enhancers were also considered in selecting methylation loci for each gene? If enhancers were not included (potentially due to the lack of coverage on the TCGA hm450 data), could the authors consider adding this as a further perspective in the discussion. This is briefly mentioned in line 371, but with the increased use of Whole genome Bisulfite sequencing (WGBS) studies in cancer, a note on the *utility of the novel approach with respect to enhancers* would be very useful. For instance, an event identified as partial mediation using only promoter CpG sites, may be tagged as fully mediated if enhancers/ transcription factor binding are considered as well.

**Minor revisions**

1. On line 251, 37 *methylation CpG sites* are reported to be associated with overall survival time of cancer. Could the authors clarify whether these are in fact *individual (non-unique) methylation sites* OR *groups (non-unique) of methylation sites.* The next few lines indicate the latter.
2. Could the authors clarify the term cancer-specific methylation sites used on line 260? Does this mean specific for a *cancer type*, in which case could the authors consider using the term ‘cancer type-specific’ or ‘cancer organ-specific’. Or does cancer-specific indicate that the relationships are observed exclusively in tumours (versus normal tissues)? Is the usage of the term consistent across the manuscript?
3. Could the authors comment on the overlap of the 57 (30 unique) methylation groups that have *total* influence on survival versus the 69 (41 unique) with *direct* influence on survival?
4. On line 325, the authors highlight the fact that nearly all the methylation-expression mediation associations identified under IUSMMT, are full mediations. However, could the authors comment on how likely it is to find partial mediations since construction of the IUSMMT does not consider the *direct* influence of methylation on survival.
5. There are some typos and grammatical errors present.

REFERENCES

Zhou, W. *et al.* DNA methylation loss in late-replicating domains is linked to mitotic cell division. *Nature genetics* **50**, 591–602 (2018).

Meir, Z., Mukamel, Z., Chomsky, E., Lifshitz, A. & Tanay, A. Single-cell analysis of clonal maintenance of transcriptional and epigenetic states in cancer cells. *Nat Genet* **52**, 709–718 (2020).
