# Supplementary material for: IUSMMT: Survival mediation analysis of gene expression with multiple DNA methylation exposures and its application to cancers of TCGA
Source: PLoS Comput Biol. 2021 Aug 31;17(8):e1009250. doi: 10.1371/journal.pcbi.1009250 (PMC8437300; doi:10.1371/journal.pcbi.1009250)
Supplement: Attachment — Submitted filename: review_PCOMPBIOL-D-21-00050.docx [file pcbi.1009250.s020.docx]

Overall, this is a strong manuscript which address the need for statistical approaches that connect regulation at multiple molecular level to clinical outcomes. This work contributes to our understanding of how epigenetic dysregulation in cancer is realized.

The authors present a statistical model which links changes in DNA methylation to survival through alterations in transcriptional regulation. They place their approach in the context of prior work and justify their modeling choices – such as integrating separate models through the intersection union and a mixture adjusted null – which were necessary to overcome challenges specific to this application. The IUSMMT model provides a general framework for applying mediation analysis to multi-omic data that is not overly complex and is well supported through simulation studies and application to TCGA. At the same time the authors make reasonable conclusions and discuss the limitations of their approach. With some improvements this paper will be a good addition to PLOS Computational Biology.

Some necessary changes are, however, required:

1. The authors need to go an extra step to establish the “functional role of DNA methylation and gene expression on cancer” by either highlight the mechanistic or translation implications of the findings. This can be accomplished by highlighting more relevant genes with literature support, discussing why CpG changes may be pleotropic while the mediating genes are cancer specific, or placing altered DNA methylation in the context of the CpG density or genomic regions.
2. Justify the inclusion of the IUSMMT-A model or do not include in the manuscript. Although the mathematic formulation in the asymptotic case is clear this model does not seem to provide any improvement over IUSMMT-E either in terms of statistical power (Fig 3) or sensitivity when identifying relevant genes (page 19).
3. Describe what data processing was applied to the DNA methylation and gene expression data in the Methods. I assume the authors are using the processed data from TCGA as is, but it would still be helpful to know what batch correction or normalization was applied if any. *(As an aside sometimes analysis with DNA methylation beta values benefit from logit transformation, see Du et al., Comparison of Beta-value and M-value methods for quantifying methylation levels by microarray analysis, 2010, BMC Bioinformatics.)*

The paper could also benefit from a few minor changes:

1. S2 Figure best illustrates why the standard IUT model is too conservative, more clearly than Table 1. Consider including this in the main text.
2. Make some of the details easier to find. For example, on page 10 reference that the rationale behind selecting simulation scenarios can be found in the methods. And on page 8 either briefly describe the asymptotic properties as sample size increases and power approaches 1 or allude to methods section.
3. Alter some of the language and word selection to improve clarity. For example, the authors should avoid using words with overloaded meaning in biological sciences like “pathway” (page 4, 7, 8, 19, and 24) when pathway is traditionally used in the context of signaling pathways, metabolic pathways, etc.
4. In the caption of Fig 4 describe the significance of the colors of the Upset Plot for consistency.
