# Supplementary material for: IUSMMT: Survival mediation analysis of gene expression with multiple DNA methylation exposures and its application to cancers of TCGA
Source: PLoS Comput Biol. 2021 Aug 31;17(8):e1009250. doi: 10.1371/journal.pcbi.1009250 (PMC8437300; doi:10.1371/journal.pcbi.1009250)
Supplement: Attachment — Submitted filename: 2021-05-28 Comments and Responses.docx [file pcbi.1009250.s021.docx]

**Point-to-points responses for reviewers’ comments**

**Reviewer: 1**

The authors have developed a novel gene-centric approach called IUSMMT (Intersection-Union Survival Mixture-adjusted Mediation Test) to delineate causal genes that mediate the effect of DNA methylation on cancer-specific survival. ISUMMT fits an empirical three-component mixture null distribution, and simulation studies demonstrated that it is higher powered than traditional approaches such as IUT (Intersection-Union Test) that rely on the uniform null distribution. The authors applied IUSMMT to 10 TCGA cancer types and revealed multiple genes (that were largely cancer-type specific) that exhibited mediation effects on cancer survival risk through the influence of altered DNA methylation at multiple CpG sites.

My overall impression is that the manuscript is a matter of interest since the new approach (IUSMMT) has potential utility in understanding the mechanism underlying the regulatory role of DNA methylation in cancer progression; and can aid in molecular classification as well as generate new insights into potential therapeutic targets. The analysis is clearly described, and the novel approach looks promising. However, I see some limitations that need addressing; and a few details that could be improved.

Thank you for your helpful and constructive comments. We have thoroughly modified our manuscript following these suggestions. We would like to thank you again for taking your valuable time to review our revised manuscript. Our detailed responses are listed below.

**Major** **Comments**

1 - The IUSMMT framework relies on the assumption that the methylation levels at CpG sites located within a gene can regulate the expression level in tumors, which in turn has an influence on patient survival. Certainly, noteworthy examples exist such as BRCA1 promoter hypermethylation in ER- breast cancer. However, recent methylation studies in large cancer datasets (Meir, et al., 2020; Zhou, et al., 2018) have illustrated that a bulk of methylation changes observed in cancer are a consequence of global epigenetic remodeling machineries including i) global loss via replication related errors; ii) CpG island/ shore methylation gain associated with tumor proliferation. In such cases, altered expression of a handful of key genes would have an in-trans effect on DNA methylation at multiple (thousands) CpG sites over the cancer epigenome; and expression of the same key genes may also have a direct effect on survival.

However, under the mediation framework described in the manuscript, the above-mentioned trends would also result in an $alpha \neq0$ as well as $beta \neq0$ ($alpha$ and $beta$ are as defined in the manuscript). Following this result, one may incorrectly assume that this is an example of the expression of a gene the influence of DNA methylation on survival, when in fact, the gene expression may itself be driving the survival, and the altered DNA methylation are just passenger events. Since a large number of methylation loci are under the influence of such epigenetic machineries (particularly proliferative gain at CpG rich promoters, that are extensively covered by the hm450 array used for the 10 TCGA datasets), could the authors consider adding this state explicitly in the model to distinguish passenger methylation events from methylation events that are likely to have a direct/ or indirect (via expression mediation) influence on survival.

Thank you for this comment. We fully agree this is an important and constructive point from both the biological and statistical perspectives. Indeed, our mediation work, like many other high-dimensional epigenetic mediation studies, relies on a key assumption that methylation CpG sites can regulate gene expression, which in turn affects the survival risk of cancers. Although supported by many prior studies [1-11], this assumption would be violated as you mentioned that bulk of methylation alterations observed in cancers might be a consequence of global epigenetic remodeling mechanism [12,13]. That is, changed DNA methylations are just passenger events, and it does not have a mediation association path from methylations to gene expression and to survival of cancers.

Therefore, as you suggested, distinguishing passenger methylation events from mediation methylation events that are likely to have a direct or indirect (via expression mediation) influence on the survival risk of cancers becomes very critical for the biological interpretation of results in our mediation analysis. To this aim, we assumed that gene expression may affect a set of DNA methylations with the effects of expression on DNA methylations following a normal distribution and performed an *ad-hoc* reverse analysis by regressing DNA methylations on expression for all the identified mediating genes based on a multivariate model. In terms of the result of the multivariate variance-component score test conducted within MultiSKAT [14], we found that approximately 16.3% (=88/543) of the discovered mediation methylation events might be passenger methylation events after the adjustment with Bonferroni’s method across these cancers.

We now added the above analysis and description in lines 392-415 on pages 19-20 and the last column of Table 2. We also further discussed this important issue in the section of discussion in lines 486-501 on pages 23-24.

2 - Could the authors elucidate the direction of the methylation alteration and gene expression change that is associated with worse survival? For example, how many were hypermethylation associated with repression?

Thank you for this comment. Following the suggestion, we added the direction of the methylation-expression effect as well as the expression-survival effect. We found that the expression level for most of the genes (68.7%=373/543) were inhibited by methylation alterations; the down-regulated expressions may lead to higher (43.4%=162/373) or lower (56.6%=211/373) survival risk of cancer patients. The expression level of some genes (~31.3%) was also upregulated by methylation alterations, likely resulting in higher (30.6%=52/170) or lower (68.8%=118/170) survival risk of cancer patients. Totally, 48.4% of (=263/543) genes have the methylation-expression effect and the expression-survival effect in the same direction and 51.6% (=280/543) of genes have opposite directions. These new results were added in lines 373-385 on pages 18-19 and Table 4.

3 - Following from the previous 2 points, could the authors also indicate whether the genes that were induced (de-repressed) included proliferation or cell cycle related genes?

Thank you for this comment. Our current work offered a novel framework for high-dimensional survival mediation analysis and can powerfully identify promising regions in which genes may act as an active mediator from methylation CpG sites to the survival risk of cancers. However, these identified genes cannot include proliferation or cell cycle related genes. Following the suggestion, we now clearly pointed out this issue in lines 459-462 on page 22.

4 - A large number of methylation-expression relationships observed in tumors are seen in normal tissues as well (but at a lower magnitude, e.g., field effects). Can the authors indicate how many of the expression-methylation relationships (identified under $alpha \neq0;$and identified under the IUSMMT) were also observed in TCGA normal tissues? The association with survival need not be investigated for normal tissues, so only comparisons in the expression-methylation relationship are requested.

Thank you for this comment. Following your suggestion, we also performed the similar methylation-expression analysis in 193 normal tissues combined across these cancers and find that 43.9% (=6075/13840) of genes are methylation-regulated (FDR < 0.05). This proportion is relatively smaller than that obtained from the tumor tissues, a direct consequence of low power due to smaller sample size. On average, 81.0% of methylation-regulated genes discovered in normal tissues are overlapped with these detected in tumor tissues across the cancers (S5A Fig). Interestingly, and as you also mentioned, we observe that the effect of methylation on expression is much stronger in tumor tissues compared with that in the normal tissue. For example, the median value of τ_2_, which can be employed to quantify the magnitude of the methylation effect on expression, is 3.7 times higher in the BRCA tissue than that in the normal tissue (S5B Fig). We now added these results in lines 271-282 on page 14.

5 - In the gene-centric model, can the authors clarify how methylation loci were selected per gene? In the methods section under “Application to ten TCGA cancer datasets”, it is indicated that UCSC Xena was used for position annotation. Was this based on a standardised promoter definition or for does it span the entire gene? The authors make the assumption that all DNA methylation CpG sites had an influence on the gene expression, and as pointed out in the discussion, this may not hold realistically. Testing the sparse relationship may be out of the scope of the manuscript, and so in the absence of this, could the authors consider performing a sensitivity analysis to fine-tune the standardised definition of methylation loci selected for the genes (for example, identifying the most efficient distance to use around the promoter?).

Thank you for this comment. We first apologize for not clearly describe how to select methylation loci in our prior manuscript. Following the suggestion, we added the quality control process for selecting methylation loci per gene. In brief, we removed non-CpG sites (probes with *ch* labels) and CpG sites located on sex chromosomes; we also excluded cross-reactive probes as suggested in [15]. The beta values of methylations were logit-transformed to obtain the M-value for each CpG locus as the M-value is not bounded between zero and one and is thus more valid for our subsequent statistical analysis [16]. We yielded methylation-expression pair for each gene in terms of the position annotation file provided by UCSC Xena. In each gene pair, we standardized both DNA methylation and expression level. According to the TCGA gene annotation mapping file, we defined in our analysis methylations as those within located within the entire gene body and 500 bps upstream of the transcription start site (TSS) so that the promoter can be included. We added these descriptions in lines 698-703 on page 34 and lines 711-721 on pages 34-35.

The distance of 500 bps is to some extent arbitrary and empirical. In fact, the optimal extension distance is not clear and various extension distances were applied in prior literature [17]. Following the suggestion, to examine the robustness of various extension distances, we performed a sensitivity analysis by incorporating methylations within distinct extended regions, and found that the extension of the distance upstream of the TSS in defining methylation loci seems to be very robust and has little influence on our final identification of mediating genes. We added these results in S8 Fig and the description in lines 711-721 on pages 34-35.

6 - Could the authors indicate whether distal enhancers were also considered in selecting methylation loci for each gene? If enhancers were not included (potentially due to the lack of coverage on the TCGA hm450 data), could the authors consider adding this as a further perspective in the discussion. This is briefly mentioned in line 371, but with the increased use of Whole genome Bisulfite sequencing (WGBS) studies in cancer, a note on the utility of the novel approach with respect to enhancers would be very useful. For instance, an event identified as partial mediation using only promoter CpG sites, may be tagged as fully mediated if enhancers/transcription factor binding are considered as well.

Thank you for this comment. We fully agree that this is an important issue from the biological perspective and it is also important for model construction. However, as you mentioned and implied in our results shown in comment #5 above, we indeed did not include methylations in distal enhancers in our analysis due to the lack of coverage on the TCGA HM450. Following your suggestion, we considered this as an interesting issue in the discussion section (please see lines 447-459 on page 22).

**Minor** **Comments**

7 - On line 251, 57 methylation CpG sites are reported to be associated with overall survival time of cancer. Could the authors clarify whether these are in fact individual (non-unique) methylation sites OR groups (non-unique) of methylation sites. The next few lines indicate the latter.

Thank you for this comment. We first apologize for the confusion. Here, the 57 associations, which were identified to be related to the overall survival time of cancer, in fact denote the discovered genes rather than methylation CpG sites. More specifically, we found there were 57 regions (i.e., genes) in which a set of methylation CpG sites defined in terms of gene were collectively associated with the overall survival time of cancer. Thus, there these 57 regions of methylation CpG sites indicated groups (non-unique) of methylation sites. Following this suggestion, we revised it as “57 (30 unique) regions of methylations at CpG sites …”. (Please see lines 244-246 on pages 11-12).

8 - Could the authors clarify the term cancer-specific methylation sites used on line 260? Does this mean specific for a cancer type, in which case could the authors consider using the term ‘cancer type-specific’ or ‘cancer organ-specific’. Or does cancer-specific indicate that the relationships are observed exclusively in tumors (versus normal tissues)? Is the usage of the term consistent across the manuscript?

Thank you for this comment. We apologize for this confusion. Following the suggestion, we now applied the term “cancer type-specific” throughout our manuscript.

9 - Could the authors comment on the overlap of the 57 (30 unique) methylation groups that have total influence on survival versus the 69 (41 unique) with direct influence on survival?

Thank you for this comment. We find that there are 12 methylation regions that not only have non-zero total effect on the survival risk, but also have substantial direct influence on the survival risk of cancers. Following the suggestion, we now added this description in lines 296-299 on page 15.

10 - On line 325, the authors highlight the fact that nearly all the methylation-expression mediation associations identified under IUSMMT, are full mediations. However, could the authors comment on how likely it is to find partial mediations since construction of the IUSMMT does not consider the direct influence of methylation on survival.

Thank you for this comment. Here, it may be not described clearly in previous manuscript, which resulted in the statement that the construction of the IUSMMT does not consider the direct influence of methylation on survival. In contrast, IUSMMT considers the direct influence of methylation on survival (denoted by **γ**^DE^), as shown in equation 4 in the main text. We previously defined the partial mediation and the full mediation in lines 532-534 on page 26. According to these definitions, in our analysis, we found that almost all the associations were full mediations, with only few being partial mediations. Following this suggestion, we discussed why many of these associations were full mediations, including low power due to high censored rate in TCGA cancer datasets, misspecification for the distribution of **γ**^DE^, and truly biological path. We added these discussions in lines 442-447 on page 22.

11 - There are some typos and grammatical errors present.

Thank you for this comment. We first apologize for these errors. A colleague from the department of English at our university helped revise our manuscript. We hope the revised manuscript would have minimal errors.

**Reviewer: 2**

**Major** **Comments**

1 - The authors claim that the found genes are causal. This is highly unlikely and should be noted. The authors should likely motivate these as informative tools for highlighting regions of interest as opposed to real causal signals. In addition, the required assumptions for a causal interpretation are not mentioned until the method (referencing to S1 text line 479), these should likely be mentioned much earlier in the paper perhaps in the introduction.

Thank you for your helpful and constructive comments. We have thoroughly revised our manuscript following these suggestions. We would like to thank you again for taking your valuable time to review our manuscript. Our detailed responses are listed below.

First, following your suggestion, in our revised manuscript, we now avoid using the term “causal”. Second, we now described these required assumptions for a causal interpretation of mediation analysis much earlier, please see lines 112-126 on page 6 in the introduction section.

2 - If the proportional hazards assumption was violated, could standard approaches be used to correct for this?

Thank you for this comment. We fully agree that the proportional hazards assumption is very important for using Cox model. Using the weighted residual method proposed in [18], we found that the proportional hazards assumption held for our Cox model (*P*>0.05). Following your suggestion, we mentioned this issue in lines 549-552 on page 27 and S5 Table.

3 - How were DNA methylation markers mapped to genes? Was it solely based on distance? If so, what was the threshold?

Thank you for this comment. We apologize for not clearly describing how to select DNA methylation markers in our prior manuscript. Following the suggestion, we now added the quality control process for selecting methylation markers per gene. In brief, we yielded methylation-expression pair for each gene in terms of the position annotation file provided by UCSC Xena. In each gene pair, we standardized both DNA methylation and expression level. According to the TCGA gene annotation mapping file, we defined in our analysis methylations as those within located within the entire gene body and a 500 bps upstream of the transcription start site (TSS) so that the promoter can be included. The distance of 500 bps is to some extent arbitrary and empirical. In fact, the optimal extension distance is not clear and various extension distances were applied in prior literature [17]. Following the suggestion, to examine the robustness of various extension distances, we performed a sensitivity analysis by incorporating methylations within distinct extended regions, and fond that the extension of the distance upstream of TSS in defining methylation loci seems to be very robust and has little influence on our final identification of mediating genes. We added these results in S8 Fig and these descriptions in lines 711-721 on pages 34-35.

4 - How was DNA methylation and gene expression standardized?

Thank you for this comment. After quality control, we yielded methylation-expression pair for each gene in terms of the position annotation file provided by UCSC Xena. In each gene pair, we standardized both DNA methylation and expression level so that methylation and expression have a mean zero and variance one. Following your suggestion, we now stated this clearly in lines 700-707 on page 34.

5 - Is the data already QCed to adjust for potential batch or technical effects? The authors mention they restrict to those people of European Ancestry, was this verified through self-report or SNP data? The number of potential mediation genes seems like there is likely some inflation or unmeasured confounder in the analysis. The supplement says quality control but no further details are given.

Thank you for this comment. Yes, we performed for both expression levels and methylations. For example, for methylations, we removed non-CpG sites (probes with *ch* labels) and CpG sites located on sex chromosomes; we also excluded cross-reactive probes as suggested in [15]. The beta values of methylations were logit-transformed to obtain the M-value for each CpG locus as the M-value is not bounded between zero and one and is thus more valid for our subsequent statistical analysis [16]. For expressions, we only consider protein coding genes and removed genes with over 50% zero expressions and variances smaller than 20% quantile of expression values. In addition, we used “self-report European ancestry” when selecting individuals included in our analysis. Following your suggestion, we now stated these clearly in the first paragraph in the section of Application to ten TCGA cancer datasets (lines 693-721 on pages 34-35).

6 - Was cell type composition of the DNA methylation adjusted for? If not, it should be as it could be driving the associations.

Thank you for this comment. Indeed, we did not consider cell type composition in the analysis. The reasons why we did do this are listed as follows. First, we used the EPIC package [19] to analyze the cell types based on gene expression and draw a Pie chart here (Fig R1), it is obvious that undefined cell type accounts for the majority, indicating that most of the cell types are not immune-relevant and thus might not very useful for the survival risk of cancer patients. Second, because the cell types are calculated from gene expression, we did not consider it as covariates in the mediation model to avoid using the data twice. Third, to our knowledge, the adjustment of cell type compositions is primarily seen for DNA available from blood samples, but is little done for tumor tissues. Although there are some studies that considered cell type compositions of the DNA methylation as covariates [20,21], we followed many other studies which did not do so (e.g., [22,23]). We hope that the reviewer also agrees with our point.

However, we indeed agree with the reviewer that considering cell types in mediation analysis is very important for in-depth understanding the biological mechanism [24]. We think this is an interesting topic for future work.

Fig R1. Pie chart of cell type compositions for the ten TCGA cancers.

7 - In SFig 6 a ch DNA methylation marker is listed. Were non-cg DNAm probes left in the analysis? These are typically removed.

Thank you for this comment. Following your suggestion, we now deleted the non-cg DNA methylation probes in the dataset.

8 - In the DNA methylation data, were DNAm sites that map to the sex chromosomes removed? Were cross reactive probes removed (PMCID: PMC3592906).

Thank you for this comment. We apologize for not clearly describing this point. Yes, we removed the methylation sites located on the sex chromosomes and we also excluded cross-reactive probes as suggested in [15].

9 - In the supplement materials discussing the \lambda values it reads as if it can be set above 1, “It needs to highlight that a value of λα or λβ that is much closer to one can lead to a higher confidence to guarantee the null of or …. However, a larger λα or λβ”. Larger than 1? Or just lambda values close to 1.

Thank you for this comment. It is a good point. It needs to highlight that a value of λ_α_ or λ_β_ that is much closer to one could lead to a higher confidence to guarantee the null of $P_{a\text{,} j}$ or $P_{\beta\text{,} j}$. However, a larger λ_α_ or λ_β_ would miss many $P_{a\text{,} j}$ and $P_{\beta\text{,} j}$ which actually come from the null distribution; in contrast, a smaller value of λ_α_ or λ_β_ that is closer to zero has the risk of incorporating non-null $P_{a\text{,} j}$ or $P_{\beta\text{,} j}$. Investigating the optimal value for λ_α_ or λ_β_ is beyond the scope of our work. In the present study, for simplicity we employ λ_α_ = λ_β_ = 0.5. We now stated this point in lines 139-145 on page 8 in the Supplementary File.

10 - What is the practical guidance for researchers for \lambda? Setting equal to 0.5?

Thank you for this comment. As mentioned above, investigating the optimal value for λ_α_ or λ_β_ is beyond the scope of our work. In the present study, for simplicity we employ λ_α_ = λ_β_ = 0.5.

11 - In the supplement figures, the authors typically show the QQ-plot and then show the histogram plots for the real data application. Would be best to be consistent. Perhaps showing the QQ-plot for Supplement Figure 5?

Thank you for this comment. We now replaced S5 Fig with QQ-plot for consistence.

12 - How many genes are involved in Table 1.

Thank you for this comment. We apologize for not clearly describing this. The number of genes (i.e., mediators) was set to 10^4^. Now we stated this point clearly there (lines 211-212 on page 10).

13 - For the power in Figure 3: How many genes was this examined over? Is the power defined as the number of times the 1% of potential genes was selected at the desired TIE?

Thank you for this comment. We apologize for not clearly describing the two points. First, the number of genes (i.e., mediators) was set to 10^4^ (lines 1160-1161 on page 49). Second, yes, the power was calculated by the proportion of true positive discoveries among the true mediation effects (lines 689-690 on pages 33-34).

14 - For the 6 different simulation scenarios: Is there a better way to display this information. At the current moment feels like a bit much and hard to take in.

Thank you for this comment. We now presented these simulation scenarios in S1 Table. We hope it is now clearer.

15 - Can the authors provide some intuition on why the estimation of the composite components is so poor under the dense null case?

Thank you for this comment. In addition, we find that the estimates of some proportion parameters (e.g., *κ*_00_) are much more biased under the dense null compared to these under the sparse null. The reason may be likely due to relatively more information that can be available for estimating these non-zero proportion parameters under the sparse null compared to the dense null (e.g., 90% vs. 10%). We added this statement in lines 195-199 on page 9.

16 - The authors discuss the power (S1 Fig) prior to the TIE (S2 Fig). While S1 appears motivated by the estimation of the parameters perhaps there is a way to discuss the TIE prior.

Thank you for this comment. In the manuscript, we first discussed the power of τ_2_ and *β* (S1 Fig), and then discussed the TIE and power of the model. In order to avoid confusion, we now added two subtitles to these two parts.

17 - The discussion of gamma total seems unnecessary and distracts from the focus of the analysis, the NIE. Why the decision to include?

Thank you for this comment. We deleted some of the descriptions of γ^TE^ in the Discussion section.

18 - Is there any biological reasoning for why there are so many signals for KIRP and survival compared to the other cancers?

Thank you for this comment. We apologize for our prior errors. We now re-analyzed these datasets and revised the new result for KIRP.

**Minor** **Comments**

19 - Line 175: typo “note”, think meant not?

Thank you and we corrected this error.

20 - Line 357: DNA methylation impacting gene expression is not a novel result.

Thank you and we removed some of the descriptions there.

21 - Figure 1: Perhaps better to have two DAGs? Reads almost like \gamma^[25] is part of the same mechanism.

Thank you and we re-draw Figure 1 and hope it clearer now.

22 - “Methylations”-> This is not typically how it is referred to as. It’s usually DNA methylation at a CpG site or methylation at probe. The authors claim that the found genes are causal. This is highly unlikely and should be noted. The authors should likely motivate these as informative tools for highlighting regions of interest as opposed to real causal signals.

Thank you, we revised the issue and used the term “DNA methylation CpG sites” in the manuscript.

**Reviewer: 3**

Overall, this is a strong manuscript which addresses the need for statistical approaches that connect regulation at multiple molecular levels to clinical outcomes. This work contributes to our understanding of how epigenetic dysregulation in cancer is realized.

The authors present a statistical model which links changes in DNA methylation to survival through alterations in transcriptional regulation. They place their approach in the context of prior work and justify their modeling choices – such as integrating separate models through the intersection union and a mixture adjusted null – which were necessary to overcome challenges specific to this application. The IUSMMT model provides a general framework for applying mediation analysis to multi-omic data that is not overly complex and is well supported through simulation studies and application to TCGA. At the same time the authors make reasonable conclusions and discuss the limitations of their approach. With some improvements this paper will be a good addition to PLOS Computational Biology.

Thank you for your helpful and constructive comments. We have thoroughly revised our manuscript following these suggestions. We would like to thank you again for taking your valuable time to review our manuscript. Our detailed responses are listed below.

Some necessary changes are, however, required:

1 - The authors need to go an extra step to establish the “functional role of DNA methylation and gene expression on cancer” by either highlight the mechanistic or translation implications of the findings. This can be accomplished by highlighting more relevant genes with literature support, discussing why CpG changes may be pleiotropic while the mediating genes are cancer specific, or placing altered DNA methylation in the context of the CpG density or genomic regions.

Thank you for this comment. This is a good point. Following your suggestion, we added a new section of “Functional role of selective DNA methylation and gene expression on TCGA cancers” to highlight the mechanistic and translation implications of the findings (lines 336-372 on pages 17-18). We described the functional role of some selective mediating genes from prior literature and discussed the implications of CpG changes may be pleiotropic while the mediating genes are cancer specific (lines 432-441 on pages 21-22).

2 - Justify the inclusion of the IUSMMT-A model or do not include in the manuscript. Although the mathematic formulation in the asymptotic case is clear this model does not seem to provide any improvement over IUSMMT-E either in terms of statistical power (Fig 3) or sensitivity when identifying relevant genes (page 19).

Thank you for this comment. Following your suggestion, here we deleted the results related to IUSMMT-A in the revised manuscript. The removal does not change our conclusion.

3 - Describe what data processing was applied to the DNA methylation and gene expression data in the Methods. I assume the authors are using the processed data from TCGA as is, but it would still be helpful to know what batch correction or normalization was applied if any. (As an aside sometimes analysis with DNA methylation beta values benefit from logit transformation, see Du et al., Comparison of Beta-value and M-value methods for quantifying methylation levels by microarray analysis, 2010, BMC Bioinformatics.)

Thank you for this comment. We apologize for not clearly describing how to select DNA methylation markers in our prior manuscript. Following the suggestion, we now added the quality control process for selecting methylation markers per gene. In brief, we yielded methylation-expression pair for each gene in terms of the position annotation file provided by Xena. In each gene pair, we standardized both DNA methylation and expression level. According to the TCGA gene annotation mapping file, we defined in our analysis methylations as those within located within the entire gene body and a 500 bps upstream of the transcription start site (TSS) so that the promoter can be included. The distance of 500 bps is to some extent arbitrary and empirical. In fact, the optimal extension distance is not clear and various extension distances were applied in prior literature. Following the suggestion, to examine the robustness of various extension distances, we performed a sensitivity analysis by incorporating methylations within distinct extended regions, and found that the extension of the distance upstream of TSS in defining methylation loci seems to be very robust and has little influence on our final identification of mediating genes. We added these results in S8 Fig and these descriptions in lines 698-703on page 34 and lines 707-721 on pages 34-35.

**Minor Comments**

4 - S2 Figure best illustrates why the standard IUT model is too conservative, more clearly than Table 1. Consider including this in the main text.

Thank you for this comment. Following the suggestion, we now included part of the results shown in S2 Figure (in the original manuscript) in the main text; please see Figure 3 on page 14.

5 - Make some of the details easier to find. For example, on page 10 references that the rationale behind selecting simulation scenarios can be found in the methods. And on page 8 either briefly describes the asymptotic properties as sample size increases and power approaches 1 or allude to methods section.

Thank you for this comment. Following the suggestion, we now included the rationale behind selecting simulation scenarios (lines 216-217 on page 10 and lines 681-688 on page 33) and briefly described the asymptotic properties of IUSMMT as sample size increases in terms of simulations (lines 172-174 on page 8).

6 - Alter some of the language and word selection to improve clarity. For example, the authors should avoid using words with overloaded meaning in biological sciences like “pathway” (page 4, 7, 8, 19, and 24) when pathway is traditionally used in the context of signaling pathways, metabolic pathways, etc.

Thank you for this comment. Following the suggestion, we revised relevant statements (e.g., avoid the use of pathway when it might be confused) to make the presentation much clearer.

7 - In the caption of Fig 4 describe the significance of the colors of the Upset Plot for consistency.

Thank you for this comment. Following the suggestion, we revised the Upset Plot in Figure 4.

**References**

1. Momparler RL, Bovenzi V (2000) DNA methylation and cancer. J Cell Physiol 183: 145-154.

2. Busslinger M, Hurst J, Flavell RA (1983) DNA methylation and the regulation of globin gene expression. Cell 34: 197-206.

3. Deaton AM, Bird A (2011) CpG islands and the regulation of transcription. Genes Dev 25: 1010-1022.

4. Moore LD, Le T, Fan G (2013) DNA methylation and its basic function. Neuropsychopharmacology : official publication of the American College of Neuropsychopharmacology 38: 23-38.

5. Wagner JR, Busche S, Ge B, Kwan T, Pastinen T, et al. (2014) The relationship between DNA methylation, genetic and expression inter-individual variation in untransformed human fibroblasts. Genome Biol 15: R37.

6. Huang WY, Hsu SD, Huang HY, Sun YM, Chou CH, et al. (2015) MethHC: a database of DNA methylation and gene expression in human cancer. Nucleic Acids Res 43: D856-861.

7. Schübeler D (2015) Function and information content of DNA methylation. Nature 517: 321-326.

8. Das PM, Singal R (2004) DNA Methylation and Cancer. J Clin Oncol 22: 4632-4642.

9. Blake LE, Roux J, Hernando-Herraez I, Banovich NE, Perez RG, et al. (2020) A comparison of gene expression and DNA methylation patterns across tissues and species. Genome Res 30: 250-262.

10. Taylor DL, Jackson AU, Narisu N, Hemani G, Erdos MR, et al. (2019) Integrative analysis of gene expression, DNA methylation, physiological traits, and genetic variation in human skeletal muscle. Proceedings of the National Academy of Sciences 116: 10883-10888.

11. Gong J, Wan H, Mei S, Ruan H, Zhang Z, et al. (2019) Pancan-meQTL: a database to systematically evaluate the effects of genetic variants on methylation in human cancer. Nucleic Acids Res 47: D1066-D1072.

12. Zhou W, Dinh HQ, Ramjan Z, Weisenberger DJ, Nicolet CM, et al. (2018) DNA methylation loss in late-replicating domains is linked to mitotic cell division. Nat Genet 50: 591-602.

13. Meir Z, Mukamel Z, Chomsky E, Lifshitz A, Tanay A (2020) Single-cell analysis of clonal maintenance of transcriptional and epigenetic states in cancer cells. Nat Genet 52: 709-718.

14. Dutta D, Scott L, Boehnke M, Lee S (2019) Multi-SKAT: General framework to test for rare-variant association with multiple phenotypes. Genet Epidemiol 43.

15. Chen Y, Lemire M, Choufani S, Butcher D, Grafodatskaya D, et al. (2013) Discovery of cross-reactive probes and polymorphic CpGs in the Illumina Infinium HumanMethylation450 microarray. Epigenetics 8: 203 - 209.

16. Du P, Zhang X, Huang C-C, Jafari N, Kibbe WA, et al. (2010) Comparison of Beta-value and M-value methods for quantifying methylation levels by microarray analysis. BMC Bioinformatics 11: 587.

17. Zhang J, Lu H, Zhang S, Wang T, Zhao H, et al. (2021) How can gene expression information improve prognostic prediction in TCGA cancers: an empirical comparison study on regularization and mixed-effect survival models. Frontiers in Genetics 10.3389/fgene.2021.667877.

18. Grambsch PM, Therneau TM (1994) Proportional hazards tests and diagnostics based on weighted residuals. Biometrika 81: 515-526.

19. Racle J, de Jonge K, Baumgaertner P, Speiser D, Gfeller D (2017) Simultaneous enumeration of cancer and immune cell types from bulk tumor gene expression data. eLife 6.

20. Gao Y, Yang H, Fang R, Zhang Y, Goode EL, et al. (2019) Testing Mediation Effects in High-Dimensional Epigenetic Studies. Frontiers in Genetics 10: 1195.

21. Fang R, Yang H, Gao Y, Cao H, Goode EL, et al. (2020) Gene-based mediation analysis in epigenetic studies. Brief Bioinform 10.1093/bib/bbaa113.

22. Huang Y-T (2019) Genome-wide analyses of sparse mediation effects under composite null hypotheses. The Annals of Applied Statistics 13: 60-84.

23. Huang Y-T (2019) Variance component tests of multivariate mediation effects under composite null hypotheses. Biometrics 75: 1191-1204.

24. Luo X, Schwartz J, Baccarelli A, Liu Z (2020) Testing cell-type-specific mediation effects in genome-wide epigenetic studies. Brief Bioinform 10.1093/bib/bbaa131.

25. Al-Haddad BJS, Jacobsson B, Chabra S, Modzelewska D, Olson EM, et al. (2019) Long-term Risk of Neuropsychiatric Disease After Exposure to Infection In Utero. JAMA Psychiatry 76: 594-602.
